# Meditation-Based Therapies for Chronic Neuropathy: A Systematic Review and Meta-Analysis

**DOI:** 10.7759/cureus.68226

**Published:** 2024-08-30

**Authors:** Cristian I Babos, Daniel C Leucuta, Dan L Dumitrascu

**Affiliations:** 1 Second Medical Department, Iuliu Hatieganu University of Medicine and Pharmacy, Cluj-Napoca, ROU; 2 Medical Department, Prof. Dr. Octavian Fodor Regional Institute of Gastroenterology and Hepatology, Cluj-Napoca, ROU; 3 Medical Informatics and Biostatistics Department, Iuliu Hatieganu University of Medicine and Pharmacy, Cluj-Napoca, ROU

**Keywords:** pain, mental healing, mindfulness, meditation, neuropathy

## Abstract

Mind-body therapies have been found to be effective in a variety of pathologies. The purpose of this study was to evaluate the efficacy of meditation-based therapies in relieving the symptoms severity, quality of life, stress and other associated mood conditions, in individuals with chronic neuropathy of various etiologies. A systematic review of randomized controlled trials, involving adult patients with persistent peripheral neuropathy, was performed. Seven article databases were searched. A meta-analysis was conducted to assess the benefits of meditation-based therapy on symptomatology, quality of life, anxiety, depression, perceived stress, sleep quality and mindfulness score. Ten of the 1133 reviewed papers were selected for quantitative review. The meditation group had a lower standardized mean difference (SMD) score (-0.47 (95% CI: -0.97 to 0.02), p=0.062) for neuropathic pain severity score; lower anxiety scores (-2.5 (95% CI: -3.68 to -1.32), p=<0.001); lower depression scores (-1.53 (95% CI: -2.12 to -0.93), p=<0.001); lower perceived stress (-1.06 (95% CI: -3.15 to 1.04), p=0.323); higher quality of life scores (2.19 (95% CI: -0.65 to 5.03), p=0.13); lower sleep quality scores (-1.27 (95% CI: -4.22 to 1.67), p=0.397); higher mindfulness scores (6.71 (95% CI: 4.09 to 9.33), p=<0.001); and lower pain severity at 1 to 1.5 follow up (-1.75 (95% CI: -2.98 to -0.51), p=0.006). Some of the results were characterized by a substantial, statistically significant heterogeneity. Nevertheless, a major part of the results pointed in the same direction, improving symptomatology with meditation-based therapy. The studies had a risk of bias mostly regarding the measurement of the outcome, randomization process and selection of the reported result. The current study discovered that the meditation group had significantly lower pain (at 1 to 1.5 months follow-up) anxiety, and depression scores and higher mindfulness scores at the end of the interventions.

## Introduction and background

Chronic neuropathic pain is a long-term and severe condition that affects millions of individuals worldwide [1], lowering patients' quality of life and creating a challenge for healthcare practitioners [2]. For instance, conditions such as painful polyneuropathy, central post-stroke pain, chemotherapy-induced neuropathy, diabetic neuropathy, post-herpetic neuralgia, and trigeminal neuralgia can be cited as examples [3]. The prevalence of chronic neuropathy is estimated at 7% of the general population and is a serious concern in each country's public health policy [4]. In terms of therapeutic alternatives, the options are quite limited and, in many cases, only have marginal effects and are resistant to conventional treatments [5]. Recent study data indicates that focused lifestyle changes, such as aerobic exercise and changes in diet that encourage weight loss, may improve the natural course of diabetic painful neuropathy and possibly other kinds of neuropathy. Several dietary supplements and vitamins, including B vitamins, vitamin D, alpha-lipoic acid, and acetyl-L-carnitine, have been investigated and found to enhance both subjective and objective neuropathic assessment [6].

In recent years, there has been an increased interest in complementary and alternative medicine (CAM). Concerning neurological disorders, 43% of peripheral neuropathy (PN) patients and 67% of multiple sclerosis (MS) patients reported the use of at least one form of CAM in the last 12 months [7,8]. Among CAM, meditation-based interventions are a frequent choice as emerging therapies. Meditation is an ancient method and tool for mental relaxation and concentration. It is realized by maintaining a condition of relaxed attentiveness. Choosing to be aware of the mind requires awareness, which directs the focus inward. Mindfulness is a type of meditation with roots in secular Buddhist meditation and has already established a well-deserved presence in many aspects of Western life. Mindfulness is defined as being aware of one's thoughts, feelings, bodily sensations, and surroundings in the present moment. Mindfulness also entails acceptance. Acceptance entails giving conscious attention to ideas and feelings without judging them [9].

Meditation-based therapies include a variety of techniques, such as mindfulness meditation, yoga, Tai Chi, and Qigong, all of which aim to develop mental awareness, emotional balance, and relaxation [10,11]. They have shown potential in treating several types of chronic pain by modifying pain perception, lowering stress, and improving general well-being [12]. Mind-body therapies also have previously been shown to be of benefit in patients with sleep disturbances, anxiety, Parkinson's disease, cancer-related fatigue, and stress, among other chronic conditions [13-18], and chronic inflammation in general [19]. It seems these effects are mediated through various neurobiological and psychological mechanisms, including changes in brain structure and function, neurotransmitter modulation, and neuroplasticity.

Mindfulness has been shown to influence key brain regions involved in pain processing and emotional regulation, such as the anterior cingulate cortex (ACC), insula, and prefrontal cortex (PFC), which are crucial for the cognitive and affective dimensions of pain. Meditation practices are associated with increased activation and structural changes in these regions [20]. Additionally, studies have revealed that meditation decreases activation of the posterior cingulate cortex, associated with self-referential thinking [21].

Meditation-based therapies also exert their effects through the modulation of neurotransmitter systems. Studies have shown that yoga-based practices correct the underactivity of the parasympathetic nervous system (PNS) and gamma-aminobutyric acid (GABA) systems in part through stimulation of the vagus nerves, the main peripheral pathway of the PNS and reduce allostatic load [22]. In meditation-based therapies, significant positive correlations were observed between functional connectivity, serotonin, and GABA [23].

A compelling mechanism by which meditation-based therapies may alleviate chronic (neuropathic) pain is through enhancing neuroplasticity. In a well-documented review by Afonso et al. (2020) [24], it was shown that functional MRI studies revealed the involvement of brain regions related to attention, inhibition, and emotional experience, as well as the default mode networks. Also, it has been emphasized that there is undisputable strong evidence that the regular practice of meditation-based techniques leads to changes in large-scale brain networks rather than only in specific regions.

There are also certain psychological mechanisms that help patients alter their perception of pain, manage stress more effectively, and enhance their overall well-being. Here are some key psychological mechanisms through which meditation-based therapies impact chronic neuropathic pain: (1) Attention regulation - by focusing attention on the present moment, often through breathing exercises or body scans. This practice reduces the habitual focus on pain and its associated emotional distress. By shifting attention away from pain, patients can decrease the intensity of pain experience [25]. (2) Cognitive reappraisal - instead of viewing pain as a threat, patients learn to see it as a neutral or even informative experience. This shift in perception can reduce the emotional impact of pain, making it more manageable and reducing emotional suffering [26]. (3) Emotional regulation - by fostering a non-judgmental awareness of thoughts and feelings. This awareness helps individuals detach from negative emotions related to pain, such as fear, anxiety, and depression, which can exacerbate the pain experience and consequently improve pain and emotional regulation outcomes [27]. (4) Body awareness - it involves a heightened sensitivity to bodily sensations. This awareness helps patients detect early signs of tension or stress that could worsen pain. It has been found to have a regulatory effect, on how the body is used for self-regulation in daily life [28]. (5) Reduction of catastrophizing - it refers to the tendency to focus on and exaggerate the threat of pain, leading to increased pain perception and emotional distress. Mindfulness meditation helps reduce catastrophizing which correlates with reduced pain intensity [29]. (6) Self-compassion - meditation fosters self-compassion, which involves treating oneself with kindness and understanding during difficult times. This attitude helps patients with chronic pain to achieve lower pain-related fear, depression, and disability, as well as greater pain acceptance, success in valued activities, and utilization of pain coping strategies [30].

These findings provide a compelling rationale for the integration of meditation-based interventions in the management of chronic neuropathic pain. Given the limitations of current therapeutic options for chronic neuropathic pain and the growing utilization of complementary and alternative therapies, this study aimed to assess the efficacy of meditation-based therapies in patients with persistent neuropathic pain associated with different chronic disorders. We hypothesized that meditation-based therapies would lead to significant reductions in pain severity, anxiety, and depression, and improvements in quality of life and mindfulness scores compared to control interventions across various types of chronic neuropathic pain.

## Review

Materials and methods

PICOS Framework

The systematic review was carried out using a PICOS framework, with (P) patients diagnosed with chronic neuropathy, (I) receiving a mindfulness intervention, (C) compared to those who did not receive such an intervention, and (O) outcomes of interest, including primary outcomes such as pain severity and secondary outcomes such as quality of life, depression, anxiety, sleep quality, mindfulness score, and perceived stress.

Eligibility Criteria

Inclusion criteria: The inclusion criteria for this comprehensive review required studies to be randomized controlled trials (RCTs) including patients with chronic neuropathy, with the intervention being a meditation-based approach compared to a control group. Eligible studies were to provide at least one primary outcome, namely pain severity, as well as secondary outcomes including quality of life, depression, anxiety, sleep quality, mindfulness score, or perceived stress.

Exclusion criteria: Exclusion criteria included non-RCT research such as reviews, observational studies, editorials, letters to the editor, and conference abstracts, as well as studies that did not follow the above-stated PICOS framework. We focused exclusively on RCTs to ensure the highest level of evidence and minimize bias. RCTs are the gold standard in clinical research, offering greater reliability and validity by reducing confounding variables. Including non-RCTs could introduce further bias, weaken the study’s internal validity, and affect the results of the review.

Information Sources

To identify the papers of interest, the following seven databases were accessed: PubMed, EMBASE, Cochrane Database, Scopus, Web of Science, PsycheNet, and Lilacs. Reference lists of selected articles and reviews were screened to identify other articles on the topic.

Search Strategy

The search strategy included the terms neuropathy, peripheric neuropathy, neuralgia, neurodynia, nerve pain, nerve disorder, meditation, mindfulness, mindfulness-based therapy (MBT), MBCT (Mindfulness Cognitive Based Therapy), MBSR (Mindfulness-Based Stress Reduction Therapy), mind-body therapies, mental healing, yoga, faith healing, spiritual healing, randomized controlled trial, along with MeSH terms, synonyms, singular and plural forms, abbreviations, as well as the Cochrane recommended search strategy for RCTs [31]. The search was performed from inception to May 3, 2024. No language restrictions were used in the search strategies nor in article selection. The complete search strategy for each database is presented in Table 1.

**Table 1:** Search strategies for different databases

PubMed
("Neuralgia"[MeSH Terms] OR "Neuralgia"[All Fields] OR "Neuralgias"[All Fields] OR "Neuropathic Pain"[All Fields] OR "Neurodynia"[All Fields] OR "Nerve Pain"[All Fields] OR (("neuralgic"[All Fields] OR "neurologic"[All Fields] OR "neurological"[All Fields] OR "neuropathic"[All Fields]) AND ("pain"[MeSH Terms] OR "pain"[All Fields])) OR ("Neuropathy"[All Fields] AND "painful"[All Fields]) OR ("Peripheral"[All Fields] AND ("neuropathy"[All Fields] OR "neuralgia"[All Fields] OR "nerve disease"[All Fields] OR "nerve disorder"[All Fields]))) AND ( ("meditation"[MeSH Terms] OR "meditation"[All Fields] OR "meditations"[All Fields] OR "meditation's"[All Fields] OR "meditational"[All Fields] OR "meditative"[All Fields] OR "meditator"[All Fields] OR "meditators"[All Fields] OR "meditate"[All Fields] OR "meditated"[All Fields] OR "meditating"[All Fields] OR "spiritual therapy"[All Fields] OR "spiritual healing"[All Fields] OR "prayer"[All Fields]) OR ("mindfulness"[MeSH Terms] OR "mindfulness"[All Fields] OR "mindful"[All Fields] OR "Self-Compassion"[MeSH Terms] OR "MBT"[TIAB] OR "MBCT"[TIAB] OR "MBSR"[TIAB]) OR ("Mind-Body Therapies"[All Fields] OR "mind-body"[All Fields] OR "mind body"[All Fields] OR "Mental Healing"[MeSH Terms] OR "mental healing"[All Fields] OR "Faith Healing"[MeSH Terms] OR "yoga"[MeSH Terms] OR "yoga"[All Fields]) ) AND ((randomized controlled trial [pt] OR controlled clinical trial [pt] OR randomized [tiab] OR placebo [tiab] OR clinical trials as topic [mesh:noexp] OR randomly [tiab] OR trial [ti]) NOT (animals [mh] NOT humans [mh]))
EMBASE
(('neuralgia'/exp OR 'neuralgia' OR 'neuralgias' OR 'neuropathic pain'/exp OR 'neuropathic pain' OR 'neurodynia'/exp OR 'neurodynia' OR 'nerve pain'/exp OR 'nerve pain' OR (('neuralgic' OR 'neurologic' OR 'neurological' OR 'neuropathic') AND ('pain'/exp OR 'pain')) OR 'polyneuropathy' OR 'peripheral neuropathy'/exp OR (('neuropathy'/exp OR 'neuropathy') AND 'painful') OR ('peripheral' AND ('neuropathy'/exp OR 'neuropathy' OR 'neuralgia' OR 'nerve disease' OR 'nerve disorder'))) AND (('meditation'/exp OR 'meditation' OR 'meditations' OR 'meditational' OR 'meditative' OR 'meditator' OR 'meditators' OR 'meditate' OR 'meditated' OR 'meditating' OR 'spiritual therapy' OR 'spiritual healing' OR 'prayer') OR ('mindfulness'/exp OR 'mindfulness' OR 'mindful' OR 'self compassion'/exp OR 'MBT':ti,ab OR 'MBCT':ti,ab OR 'MBSR':ti,ab) OR ('mind-body therapy' OR 'mind-body therapies' OR 'mind-body' OR 'mind body' OR 'mental healing' OR 'faith healing'/exp OR 'spiritual healing'/exp OR 'yoga'/exp OR 'yoga')) ) AND (('randomized controlled trial'/exp OR 'controlled clinical study'/exp OR random$:ti,ab OR 'randomization'/exp OR 'intermethod comparison'/exp OR placebo:ti,ab OR compare:ti OR compared:ti OR comparison:ti OR ((evaluated:ab OR evaluate:ab OR evaluating:ab OR assessed:ab OR assess:ab) AND (compare:ab OR compared:ab OR comparing:ab OR comparison:ab)) OR (open:ti,ab AND adj:ti,ab AND label:ti,ab) OR ((double:ti,ab OR single:ti,ab OR doubly:ti,ab OR singly:ti,ab) AND adj:ti,ab AND (blind:ti,ab OR blinded:ti,ab OR blindly:ti,ab)) OR 'double blind procedure'/exp OR parallel) AND group$1:ti,ab OR crossover:ti,ab OR 'cross over':ti,ab OR ((assign$:ti,ab OR match:ti,ab OR matched:ti,ab OR allocation:ti,ab) AND adj5:ti,ab AND (alternate:ti,ab OR group$1:ti,ab OR intervention$1:ti,ab OR patient$1:ti,ab OR subject$1:ti,ab OR participant$1:ti,ab)) OR assigned:ti,ab OR allocated:ti,ab OR (controlled:ti,ab AND adj7:ti,ab AND (study:ti,ab OR design:ti,ab OR trial:ti,ab)) OR volunteer:ti,ab OR volunteers:ti,ab OR 'human experiment'/exp OR trial:ti) NOT ((((((random$:ti,ab AND adj:ti,ab AND sampl$:ti,ab AND adj7:ti,ab AND ('cross section$':ti,ab OR questionnaire$1:ti,ab OR survey$:ti,ab OR database$1:ti,ab) NOT ('comparative study'/exp OR 'controlled study'/exp OR 'randomi?ed controlled':ti,ab OR 'randomly assigned':ti,ab) OR 'cross-sectional study'/exp) NOT ('randomized controlled trial'/exp OR 'controlled clinical study'/exp OR 'controlled study'/exp OR 'randomi?ed controlled':ti,ab OR 'control group$1':ti,ab) OR (case:ti,ab AND adj:ti,ab AND control$:ti,ab AND random$:ti,ab NOT 'randomi?ed controlled':ti,ab) OR ('systematic review':ti NOT (trial:ti OR study:ti)) OR (nonrandom$:ti,ab NOT random$:ti,ab) OR 'random field$':ti,ab OR ('random cluster':ti,ab AND adj3:ti,ab AND sampl$:ti,ab) OR (review:ab AND review:pt)) NOT trial:ti OR 'we searched':ab) AND (review:ti OR review:pt) OR 'update review':ab OR (databases:ab AND adj4:ab AND searched:ab) OR rat:ti OR rats:ti OR mouse:ti OR mice:ti OR swine:ti OR porcine:ti OR murine:ti OR sheep:ti OR lambs:ti OR pigs:ti OR piglets:ti OR rabbit:ti OR rabbits:ti OR cat:ti OR cats:ti OR dog:ti OR dogs:ti OR cattle:ti OR bovine:ti OR monkey:ti OR monkeys:ti OR trout:ti OR marmoset$1:ti) AND 'animal experiment'/exp OR 'animal experiment'/exp) NOT ('human experiment'/exp OR 'human'/exp))
Cochrane
("Neuralgia" OR "Neuralgias" OR "Neuropathic Pain" OR "Neurodynia" OR "Nerve Pain" OR (("neuralgic" OR "neurologic" OR "neurological" OR "neuropathic") AND ("pain")) OR ("Neuropathy" AND "painful") OR ("Peripheral" AND ("neuropathy" OR "neuralgia" OR "nerve disease" OR "nerve disorder"))) AND ( ("meditation" OR "meditations" OR "meditation's" OR "meditational" OR "meditative" OR "meditator" OR "meditators" OR "meditate" OR "meditated" OR "meditating" OR "spiritual therapy" OR "spiritual healing" OR "prayer") OR ("mindfulness" OR "mindful" OR "MBT" OR "MBCT" OR "MBSR") OR ("Mind-Body Therapies" OR "mind-body" OR "mind body" OR "mental healing" OR "yoga"))
Scopus
TITLE-ABS-KEY("Neuralgia" OR "Neuralgias" OR "Neuropathic Pain" OR "Neurodynia" OR "Nerve Pain" OR (("neuralgic" OR "neurologic" OR "neurological" OR "neuropathic") AND ("pain")) OR ("Neuropathy" AND "painful") OR ("Peripheral" AND ("neuropathy" OR "neuralgia" OR "nerve disease" OR "nerve disorder"))) AND TITLE-ABS-KEY( ("meditation" OR "meditations" OR "meditation's" OR "meditational" OR "meditative" OR "meditator" OR "meditators" OR "meditate" OR "meditated" OR "meditating" OR "spiritual therapy" OR "spiritual healing" OR "prayer") OR ("mindfulness" OR "mindful" OR "MBT" OR "MBCT" OR "MBSR") OR ("Mind-Body Therapies" OR "mind-body" OR "mind body" OR "mental healing" OR "yoga")) AND ("randomized controlled trial" OR ("randomized" AND "controlled" AND "trial") OR TITLE-ABS-KEY(randomized) OR TITLE-ABS-KEY(placebo) OR ("clinical" AND ("trial" OR "trials")) OR TITLE-ABS-KEY(randomly) OR TITLE(trial)) AND NOT (TITLE-ABS-KEY(rat) OR TITLE-ABS-KEY(rats) OR TITLE-ABS-KEY(mouse) OR TITLE-ABS-KEY(mice) OR TITLE-ABS-KEY(swine) OR TITLE-ABS-KEY(porcine) OR TITLE-ABS-KEY(murine) OR TITLE-ABS-KEY(sheep) OR TITLE-ABS-KEY(lambs) OR TITLE-ABS-KEY(pigs) OR TITLE-ABS-KEY(piglets) OR TITLE-ABS-KEY(rabbit) OR TITLE-ABS-KEY(rabbits) OR TITLE-ABS-KEY(cat) OR TITLE-ABS-KEY(cats) OR TITLE-ABS-KEY(dog) OR TITLE-ABS-KEY(dogs) OR TITLE-ABS-KEY(cattle) OR TITLE-ABS-KEY(bovine) OR TITLE-ABS-KEY(monkey) OR TITLE-ABS-KEY(monkeys) OR TITLE-ABS-KEY(trout)) AND ( LIMIT-TO ( DOCTYPE , "ar" ) OR LIMIT-TO ( DOCTYPE , "re" ) OR LIMIT-TO ( DOCTYPE , "le" ) ) AND ( LIMIT-TO ( SUBJAREA , "MEDI" ) ) AND ( LIMIT-TO ( SRCTYPE , "j" ) ) AND ( LIMIT-TO ( EXACTKEYWORD , "Human" ) OR LIMIT-TO ( EXACTKEYWORD , "Humans" ) )
Web of Science
TS=(("Neuralgia" OR "Neuralgias" OR "Neuropathic Pain" OR "Neurodynia" OR "Nerve Pain" OR (("neuralgic" OR "neurologic" OR "neurological" OR "neuropathic") AND ("pain")) OR ("Neuropathy" AND "painful") OR ("Peripheral" AND ("neuropathy" OR "neuralgia" OR "nerve disease" OR "nerve disorder"))) AND ( ("meditation" OR "meditations" OR "meditation's" OR "meditational" OR "meditative" OR "meditator" OR "meditators" OR "meditate" OR "meditated" OR "meditating" OR "spiritual therapy" OR "spiritual healing" OR "prayer") OR ("mindfulness" OR "mindful" OR "MBT" OR "MBCT" OR "MBSR") OR ("Mind-Body Therapies" OR "mind-body" OR "mind body" OR "mental healing" OR "yoga")))
Lilacs
tw:(("Neuralgia" OR "Neuralgias" OR "Neuropathic Pain" OR "Neurodynia" OR "Nerve Pain" OR (("neuralgic" OR "neurologic" OR "neurological" OR "neuropathic") AND ("pain")) OR ("Neuropathy" AND "painful") OR ("Peripheral" AND ("neuropathy" OR "neuralgia" OR "nerve disease" OR "nerve disorder"))) AND ( ("meditation" OR "meditations" OR "meditation's" OR "meditational" OR "meditative" OR "meditator" OR "meditators" OR "meditate" OR "meditated" OR "meditating" OR "spiritual therapy" OR "spiritual healing" OR "prayer") OR ("mindfulness" OR "mindful" OR "MBT" OR "MBCT" OR "MBSR") OR ("Mind-Body Therapies" OR "mind-body" OR "mind body" OR "mental healing" OR "yoga")))
PsycheNet
("Neuralgia" OR "Neuralgias" OR "Neuropathic Pain" OR "Neurodynia" OR "Nerve Pain" OR (("neuralgic" OR "neurologic" OR "neurological" OR "neuropathic") AND ("pain")) OR ("Neuropathy" AND "painful") OR ("Peripheral" AND ("neuropathy" OR "neuralgia" OR "nerve disease" OR "nerve disorder"))) AND ( ("meditation" OR "meditations" OR "meditation's" OR "meditational" OR "meditative" OR "meditator" OR "meditators" OR "meditate" OR "meditated" OR "meditating" OR "spiritual therapy" OR "spiritual healing" OR "prayer") OR ("mindfulness" OR "mindful" OR "MBT" OR "MBCT" OR "MBSR") OR ("Mind-Body Therapies" OR "mind-body" OR "mind body" OR "mental healing" OR "yoga"))

Selection Process

First, a semiautomated elimination of duplicate studies was performed in Zotero, produced by the Corporation for Digital Scholarship (Vienna, Virginia, USA) [32]. The remaining references were handled also with Zotero. Then, two authors (CIB and DCL) manually screened the title and abstracts, excluded articles that did not meet the selection criteria, and duplicated studies. Disagreements were solved by discussion. Next, the same authors manually selected articles among the retrieved full-text versions of the remaining articles, excluding irrelevant articles, articles of a different intended type, or duplicate studies. Disagreements were solved by discussion.

Data Collection Process and Data Items

From each selected paper, data was manually extracted by one author (CIB) in a Microsoft (Redmond, Washington, USA) Office 365 Excel file, concerning the characteristics of the study, country, region, trial design, exposure duration, study population, age, gender, intervention, control intervention, outcomes, as well as the endpoints, pain severity, including secondary outcomes such as quality of life, anxiety, depression. Next, another author (DCL) rechecked the extracted data with the content of the full-text paper.

Study Risk of Bias Assessment

All the selected studies were assessed for the presence of bias using the Cochrane Risk of Bias tool 2 [33] by two authors (CIB and DCL). Discrepancies were addressed through in-depth discussions between the two authors to investigate the underlying reasons for differing evaluations of bias risk. The authors carefully reviewed the relevant criteria and theory from the risk of bias measurement instrument and reread the necessary sections of the full article until a consensus was reached.

Effect Measures

We identified four outcome measures of interest that we assessed in our analyses: pain intensity, quality of life, anxiety assessment, depression, perceived stress, sleep quality, and mindfulness score assessment. For each outcome, the mean and standard deviation were extracted and converted to standard errors. In cases where these data were missing, data were computed from confidence intervals (CIs) using formulas from the Cochrane Handbook [34]. The values of interest were represented by differences in the changes or differences in the final measurements. The measurements were extracted at the end of the study and for follow-up. The effect measure of interest was the standardized mean difference (SMD) along with its standard error.

Synthesis Methods

The mean differences and standard errors were subjected to meta-analyses using the meta package [35]. Because of the clinical heterogeneity (differences in study populations, interventions, and outcomes) between the trials, the SMD (a statistical measure that allows comparison across studies using different scales) and 95% CI for each variable were calculated using the random effects model, which accounts for variability both within and between studies. The results were presented as forest plots. To assess statistical heterogeneity between studies (the degree of variation in study outcomes), we used the chi-squared-based Q-test (a statistical test that assesses whether differences in results are due to chance) and I² (a statistic that quantifies the percentage of variation that is due to heterogeneity rather than chance). The heterogeneity was classified using the Cochrane Collaboration guide: 0%-40%: might not be important; 30%-60%: moderate heterogeneity; 50%-90%: substantial heterogeneity; 75%-100%: considerable heterogeneity [34]. The assumption of statistical significance was made if the p-value was less than 0.05. To examine the robustness of the findings, a leave-one-out sensitivity analysis was undertaken, in which one study was removed from the analysis at a time to see if any single study significantly affected the entire set of results. Subgroup analyses, which entail separating data into smaller groups based on specified criteria (in this example, various etiologies) to check if the results differ within these groups, were used to investigate the influence of different etiologies on the results. The R environment for statistical computation and graphics, version 4.3.2 [36], from the R Foundation for Statistical Computing, Vienna, Austria, was used for all analyses.

Reporting Bias Assessment

The publication bias (the tendency for studies with positive results to be published more frequently than those with negative or inconclusive results) was evaluated using funnel plots (a graphical method for detecting asymmetry that may indicate bias) and the Egger test (a statistical test that quantitatively evaluates the funnel plot's symmetry and the presence of bias).

Results

**Figure 1:**
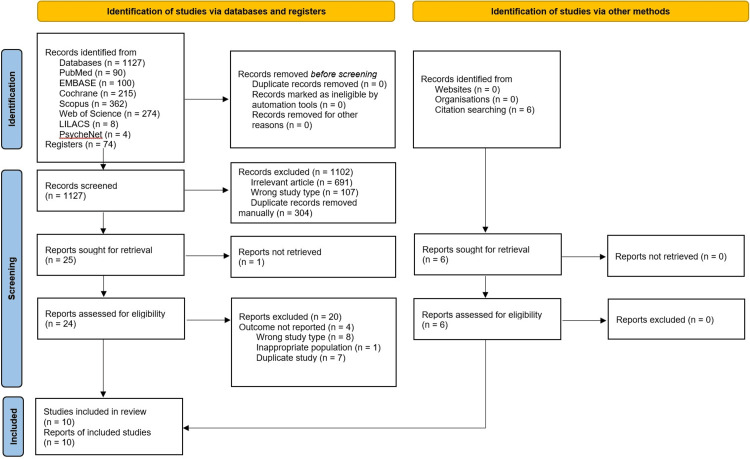
Identification, screening, and inclusion of articles flowchart (PRISMA flowchart) PRISMA: Preferred Reporting Items for Systematic Reviews and Meta-Analyses

A total of 1133 results were retrieved from seven searched databases. The identification and selection process is presented in Figure 1. After the removal of duplicates and irrelevant or wrong study types, 30 studies remained for the full-text selection assessment. Studies in which the outcome was not reported, the type of study was not a randomized controlled study, the population was inappropriate, or in case of a duplicate, were further excluded. Finally, 10 articles were included in the review and were meta-analyzed.

Study Characteristics

The study characteristics are presented in detail in Table 2. Four studies were conducted in North America (two in the USA [37,38], one in Canada [39]), one in Central America (Mexico [40]), two in Europe (one in the UK [41], one in Denmark [42]) and four in Asia (two in India [43,44], one in Iran [45], one in China [46]). All the studies used a parallel design and had an intervention length ranging from three to 12 weeks. The pathologies related to the studies had as a common denominator the chronic neuropathic pain, being as follows: chemotherapy-induced PN (four studies), post-herpetic neuropathy (two studies), and one of each included spinal cord injury, chronic orofacial pain, Guillan-Barre syndrome and MS.

**Table 2:** Study characteristics NPRS: Numerical Pain Rating Scale; PSQI: Pittsburgh Sleep Questionnaire Index; HADS: Hospital Anxiety and Depression Scale; BI: Barthel Index; WHO QOL BREF: World Health Organization Quality of Life Bref Scale; FFMQ: Five Facet Mindfulness Questionnaire; PCS: Pain Catastrophizing Scale; SF MPQ 2: Short Form McGill Pain Questionnaire 2; WHO 5: World Health Organization 5 Well Being Index; STAI: State Trait Anxiety Index; CES D: The Center of Epidemiological Studies Depression Scale; NPSI: Neuropathic Pain Symptom Inventory; BPI: Brief Pain Inventory; POMS: Profile Of Mood States; PHQ: Patient Health Questionnaire; SF 12 PS: Short Form 12 Physical Score; SF 12 MS: Short Form 12 Mental Score; VAS: Visual Analog Scale; MS QOL 54: Multiple Sclerosis Quality of Life 54; MOS SF 36: Medical Outcome Study Short Form 36; HAMD: Hamilton Depression Rating Scale; HAMA: Hamilton Anxiety Rating Scale; FACT/GOG Ntx: Functional Assessment of Cancer Therapy/Gynaecology Oncologic Group - Neurotoxicity Subscale

Study	Country	Region	Trial design	Exposure duration (weeks)	Study population	Age (years), mean (SD)/median (IQR) {range} Patient/Control	Female (%) Patient/control	Intervention	Control	Outcome main	Other outcomes	Inference
Sendhilkumar, 2013 [44]	India	Asia	parallel	3	Guillain-Barré syndrome	32.30 ± 9.911/31.30 ± 14.317	20/50	Five sessions of yoga (one hour/day)+rehabilitation therapeutics	Rehabilitation therapeutics (pharmacotherapy, physiotherapy, occupational therapy and orthotic management)	NPRS	PSQI, HADS, and functional status were recorded using BI	Yoga (including relaxation, pranayama, and meditation) significantly improved sleep quality in GBS patients compared to the control group; Both groups showed reductions in pain, anxiety, and depression, but they were not statistically significant.
Hearn JH, 2018 [41]	United Kingdom	Europe	parallel	8	Spinal cord injury	43.8 +/- 8.7/45.2 +/- 12.2	53/55	A 2 x 10 min audio-guided meditations/day (six days/week) within an online Mindfulness-Based Pain Management programme	Psycho-educational content on spinal cord injury and chronic pain, to read in an email once per week	NPRS on pain intensity, and pain unpleasantness	HADS, WHOQoL-BREF, FFMQ global score, subscales; PCS.	Internet-delivered mindfulness training can lead to greater improvements in depression, anxiety, pain catastrophizing, and specific facets of mindfulness compared to psychoeducation for people with spinal cord injury.
Johannsen, 2016 [42]	Denmark	Europe	parallel	8	Breast cancer	56.8 (9.99)/56.7 (8.10)	100	Slightly shorter two-hour sessions, shorter meditation exercises (# 30 min), more gentle yoga exercises, and omission of the whole-day session. MBCT was delivered in groups of 13 to 17 participants in weekly sessions over eight consecutive weeks.	Wait-List	NPRS, 11-item scale; SF-MPQ-2 Global, with subscales: continuous, intermittent, neuropathic, effective. Pain burden with NPRS	QoL was assessed with the WHO-5; Psychological distress was assessed with the HADS.	MBCT had a statistically significant, robust, and durable effect on reducing pain intensity in women treated for breast cancer. MBCT also had a statistically significant effect on improving quality of life and reducing the use of non-prescription pain medication.
Meize-Grochowski, 2015 [40]	Mexico	Central America	parallel	6	Postherpetic neuralgia	72 (9.6) {55-90}	46/64	One-hour individual introductory session with a certified MBSR instructor, followed by mindful meditation using a focus on breathing guided by CDs 6 to 15 minutes a day.	Usual Care	SF-MPQ-2	QoL-The RAND 36-Item Health Survey 1.0; CES-D, STAI	The meditation intervention showed promising trends in improving affective pain, physical functioning, and emotional well-being in the treatment group compared to the control group, though the differences were not statistically significant
Shergill, 2022 [39]	Canada, Ontario/Ottawa	North America	parallel	8	Breast cancer	51.3 (11.4)/55.1 (9.6)	100	A 2.5-hour weekly sessions along with a full day (approximately six hours) retreat held halfway through the course on a weekend.	Wait-List with the MBSR program participation offer, within three months from the intervention completion of the intervention group.	NPSI	BPI Short-interference subscale. The secondary outcomes included pain, emotional function, QoL, and global impression of change as informed by the IMMPACT group. increased anxiety with POMS; Patient Global Impression of Change; PHO; FFMQ global; PCS; NPSI; SF-12 PS; Short-Form-12 MS	The MBSR intervention did not result in significant improvements in other outcomes such as pain, emotional function, quality of life, and global impression of change compared to the control group.
Bhalla, 2019 [43]	India	Asia	parallel	12	Orofacial pain (myofascial pain and trigeminal neuralgia)	NR	NR	Meditation, Yoga, acupuncture, Usual care, facial massage therapy, hot and cold therapy	Usual Care	VAS	QoL Scale adopted by the American Chronic Pain Association, Stress scale adopted by the American Heart Association	Holistic approaches combined with pharmacological therapy showed better outcomes than pharmacological therapy alone for both trigeminal neuralgia and myofascial pain.
Doulatabad, 2012 [45]	Iran	Asia	parallel	12	Multiple Sclerosis	31.6 ± 8	100	The case group underwent Yoga therapy for three months, keeping the pace of eight 60 to 90 minute-lasting sessions per month	No intervention	MSQOL-54	Multiple Sclerosis QoL-54 (MSQOL-54)	Practising Yoga techniques can alleviate physical pain and improve the quality of life of multiple sclerosis (MS) patients.
Lengacher, 2021 [37]	USA	North America	parallel	6	Breast cancer	56.5/57.6	100	A two hours sessions/week, for six weeks + practice at home, 15-45 min/daily	Usual Care	BPI, pain intensity subscale	CES-D; STAI; MOS SF-36 are used to assess mental health as related to QOL; PSQI.	Reductions in fear of cancer recurrence and perceived stress mediated the positive effects of the MBSR intervention on reducing anxiety and fatigue in breast cancer survivors.
Zhu, 2019 [46]	China	Asia	parallel	8	Postherpetic neuralgia	55.2 ± 5.1 vs. 54.9 ± 4.6	44/48	Mindfulness-based stress reduction (MBSR)	Usual care	NPRS	HAMD, HAMA	MBSR can effectively reduce depression, anxiety, and pain in patients with postherpetic neuralgia. The MBSR program was effective in improving psychological and physical symptoms in patients with postherpetic neuralgia.
Bao, 2020 [38]	USA	North America	parallel	8	Breast 38 (92.7) Yoga 18 (85.7) UC 20 (100.0) Uterine 2 (4.9) 2 (9.5) 0 (0.0) Ovarian 1 (2.4) 1 (4.8) 0 (0.0)	60.0 (35.5, 77.9)/62.3 (42.4, 79.0)	100	A one-hour yoga session daily, for eight weeks	Wait-List control arm, the usual care group did not receive any intervention throughout the 12 weeks.	Numeric Pain Rating Scale (NPRS)	FACT/GOG-Ntx; the 11-item Neurotoxicity subscale of the FACT/GOG-Ntx questionnaire to assess neuropathy-related quality of life; The Functional Reach Test assesses stability and balance; Chair to stand is a standardized physical performance test, for independent living and fall prevention.	Yoga appears to be safe and shows promising efficacy in improving neuropathy symptoms and functional outcomes, with yoga improving pain, quality of life, and physical functioning outcomes compared to usual care.

The mean age of the patients in the studies was heterogenous; for those from the studies referring to chemotherapy-induced chronic neuropathy, it was around 55-60 years, composed exclusively of females [37-39,42], also in the study related to MS, which enrolled only women and the mean age was around 31 years [45]. For the other studies, the mean age maintained heterogeneous ranging from 32 to 72 years of age, yet with a quite balanced distribution based on sex [40,41,43,46]. There was an exception in the study related to Guillan-Barre syndrome, conducted in India, where the percentage of women in the intervention group was 20% [44].

Concerning the intervention program, four studies [37,39,40,46] used MBSR [47], one study [42] used Mindfulness-Based Cognitive Therapy (MBCT) [48], one study [41] used Mindfulness-Based Pain Management program [49] and four studies [38,43-45] used yoga meditation and physical postures, of different methods (Ashtanga yoga [50], mind sound relaxation technique [51], pranayama/the breath control [52]). All studies used group therapy. The sessions set up was diverse: 10 minutes daily sessions, six days a week, in one study [41], a one-hour daily session in one study [44], a one-hour introductory session in person and then 6-15 minutes a day, daily home practice in one study [40], twice a week sessions of 1-1.5 hours in two studies [38,45], weekly sessions lasting between two hours and 3.5 hours in four studies [37,39,42,46], and in one study [43], the time duration of sessions and frequency was not mentioned. One study additionally used a workshop retreat week of three- or four-hour length [39]. The training was provided by a research team member with experience with the mindfulness practice in the six studies with mindfulness-based intervention [37,38-42,46], while the four studies based on yoga did not mention explicitly that the intervention provided by a yoga instructor [33,38-45]. In two studies, the years of experience for the trained person were emphasized as being over five respectively 15 years [39,40]. Most of the studies encouraged the patients to practice at home and some of them monetarized this activity through the completion of diaries or call reminders [37,46]. The control group received the usual care. In one study [43], the patients enrolled in the control group were offered, besides conventional treatment, information materials about the disease, and psychological and social support. Four studies used a waitlist design, offering the therapy used in the intervention group, on the study completion, to the control group, too [37,38,42,46].

The inclusion criteria were in most studies clearly and extensively presented. One study [43] did not present any inclusion or exclusion criteria, except the presence of the assessed neuropathic pathology. One study [46] had age limitations besides the related disorder, and another one [40] added language conditions, too. One study [45] had age, sex, and ability to physical exercise as inclusion conditions. The other studies [37-39,41,42,44] mentioned quite a large set of criteria, like language, disease stage, off-treatment time period, the presence of neuropathic pain of a minimum certain grade and two studies [38,44] added also age limitations.

The exclusion criteria were comprehensively presented in most of the articles [37,39-42,44-46], as follows: disease stage, severe comorbid pathologies such as cardiovascular, metabolic, respiratory, epileptic, musculoskeletal and severe psychiatric disorder, cognitive impairment, previous practice of meditation based technics. One study [39] added expected survival time and one study [42] added gender, to these criteria. In one study [38], there were no exclusion conditions expressed.

Treatment Outcomes

**Figure 2:**
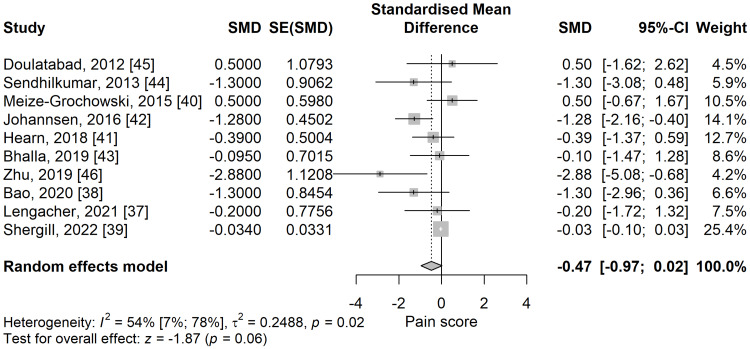
The standardized mean difference concerning the pain scores when comparing meditation intervention with control. SMD: standardized mean difference; SE: standard error of the treatment effect; CI: confidence interval

Pain score: We compared SMDs between meditation and control groups on 10 selected studies and lower pain scores were found in the meditation group compared to the control group (SMD = -0.47; 95% CI: -0.97 to 0.02; p=0.062) (Figure 2), relatively close to the significance level. The heterogeneity between the studies was substantial and statistically significant, with I^2^ of 54.5% (95% CI 7.1% to 77.7%), p=0.019.

**Figure 3:**
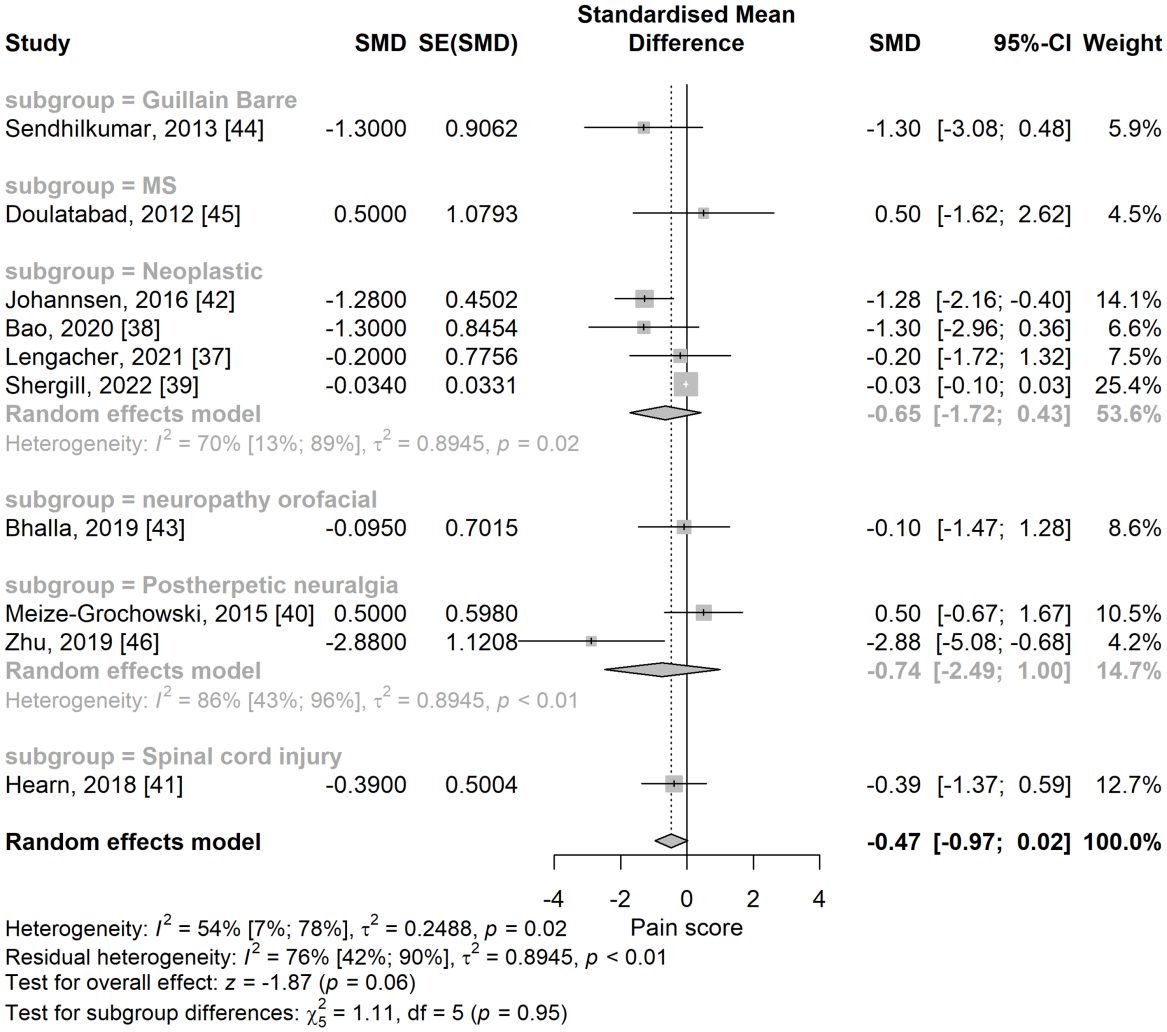
Standardized mean difference concerning the pain score, when comparing meditation intervention with control, and subgroup analyses in the function of the type of neuropathic pain. SMD: standardized mean difference; SE: standard error of the treatment effect; CI: confidence interval

Subgroup analyses based on the type of neuropathic pain did not reach significance levels (Figure 3). The largest subgroup was that of neoplastic neuropathic pain, with four studies [37-39,42] showing a reduction in pain but without being statistically significant (SMD = -0.65; 95% CI: -1.72 to 0.43).

**Figure 4:**
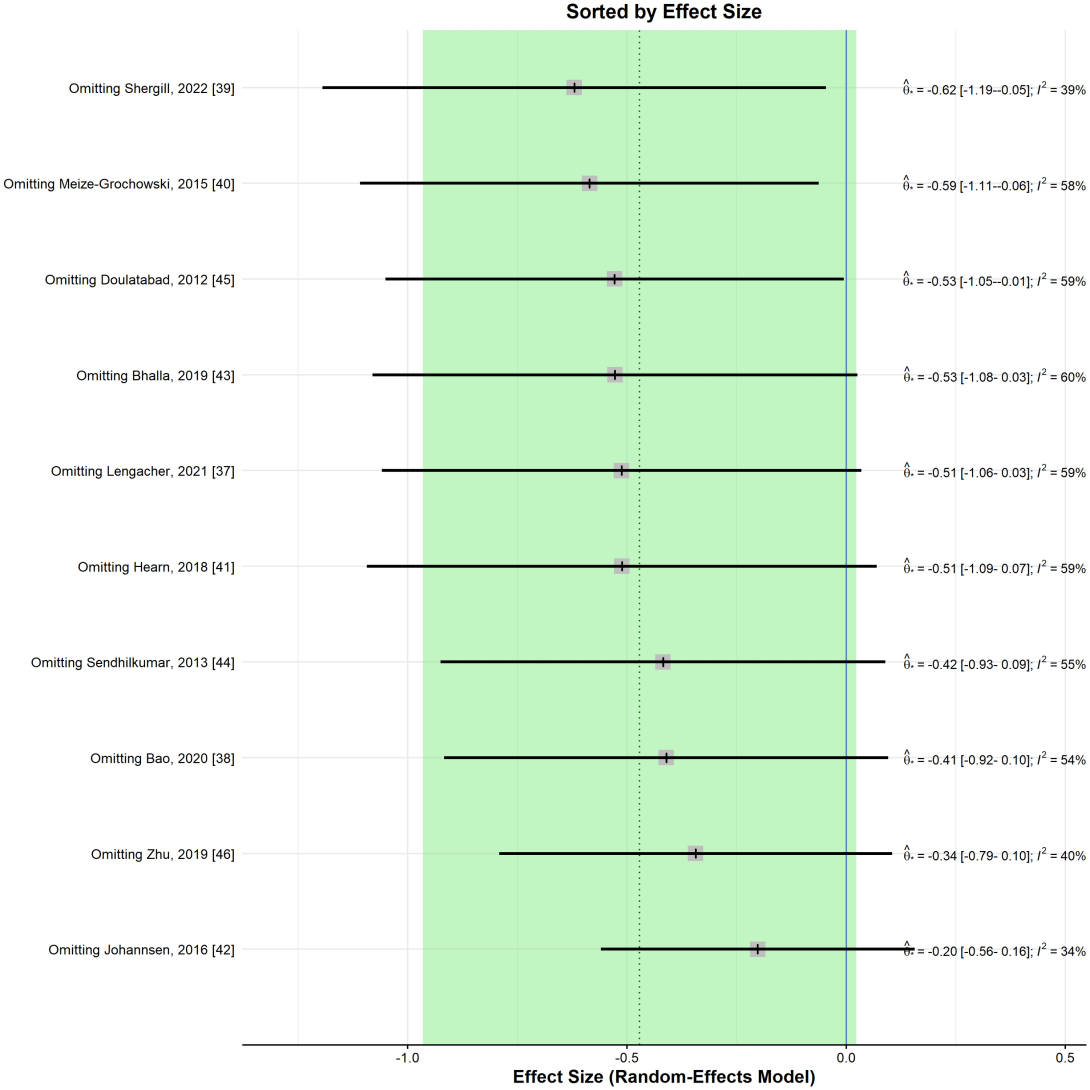
Leave-one-out sensitivity analysis plot for standardized mean difference (Θ ^[95% confidence interval]) concerning the pain score, when comparing meditation intervention with control.

Nevertheless, for all leave-one-out sensitivity analyses, the direction of the effect was in favour of the meditation group, albeit not statistically significant (Figure 4).

**Figure 5:**
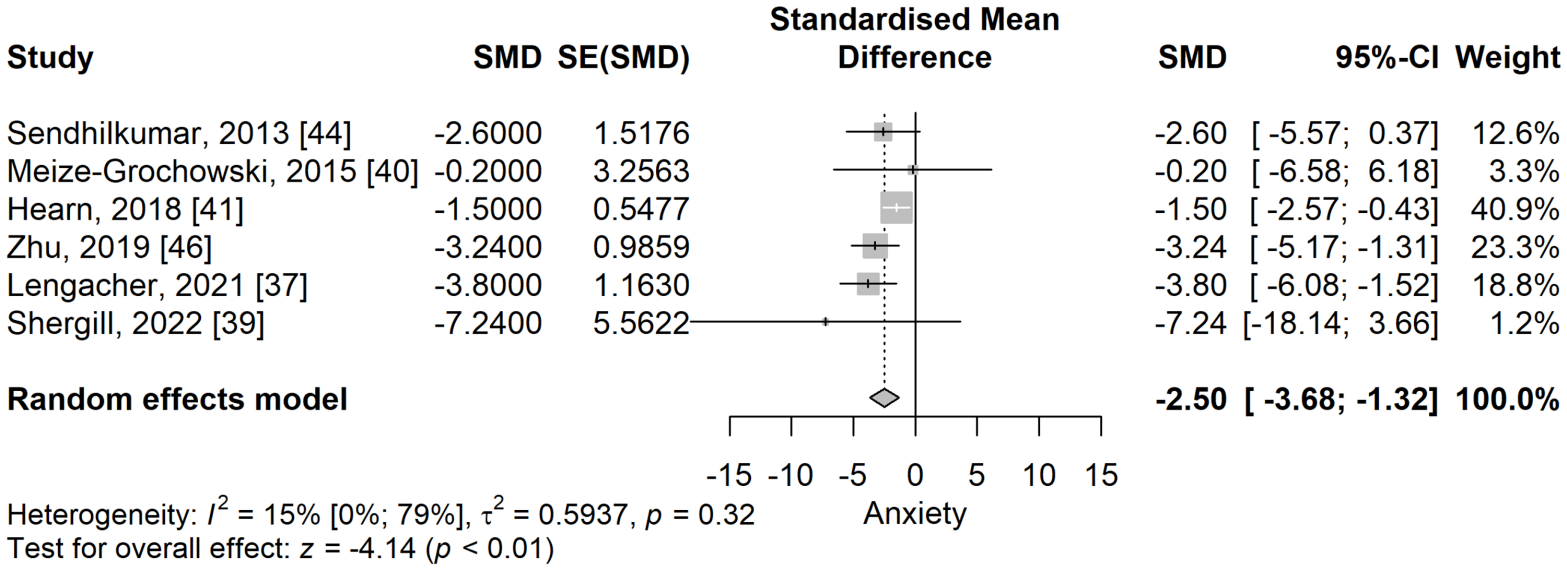
The standardized mean difference concerning the anxiety scores when comparing meditation intervention with control. SMD: standardized mean difference; SE: standard error of the treatment effect; CI: confidence interval

Anxiety: The anxiety scores were significantly lower in the meditation group compared to the control group in a meta-analysis of five studies (SMD: -2.5; 95% CI: -3.68 to -1.32; p=<0.001) (Figure 5). No significant heterogeneity was observed, I^2^ being 15.3% (95% CI 0% to 78.5%), p=0.315.

**Figure 6:**
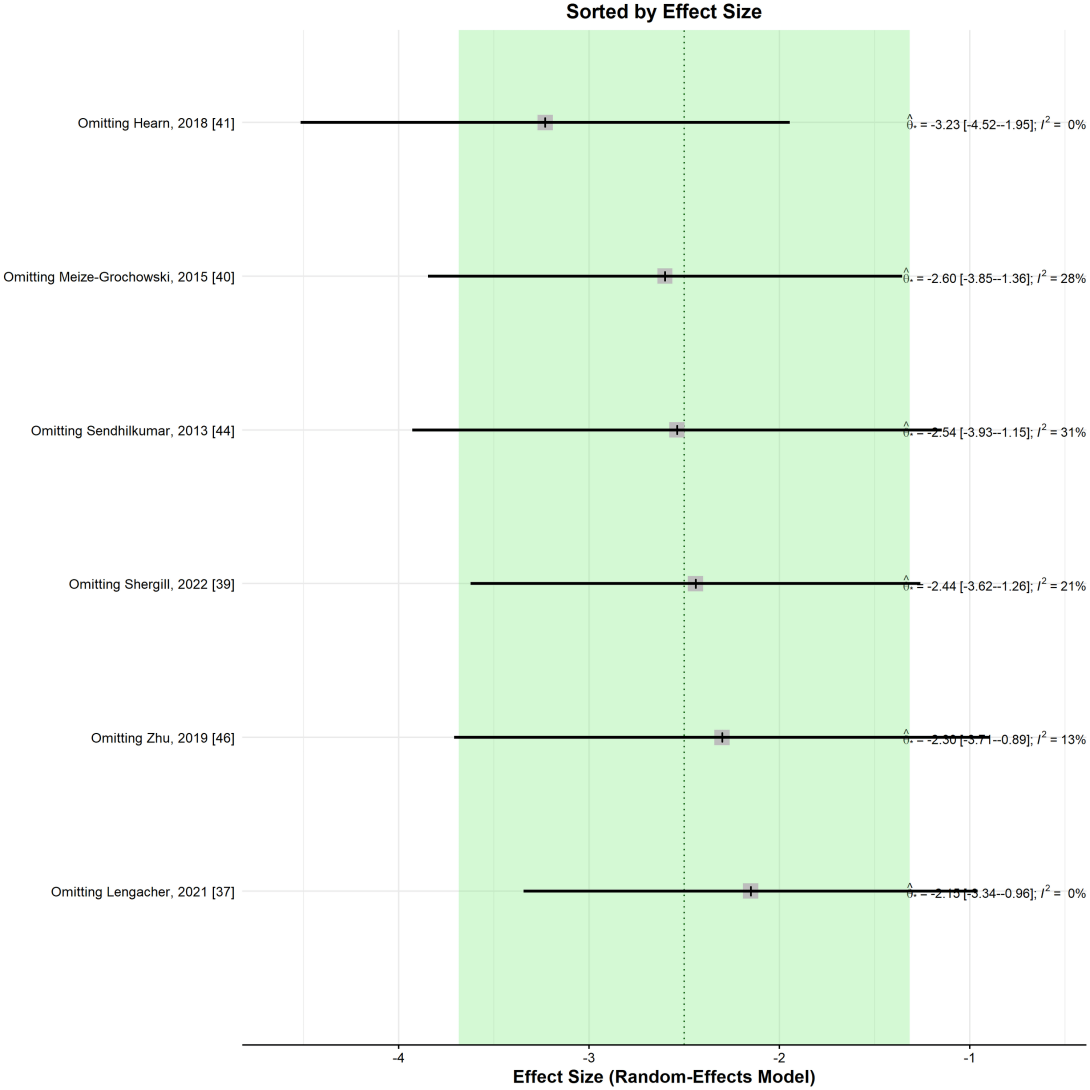
Leave-one-out sensitivity analysis plot for standardized mean difference (Θ ^[95% confidence interval]) concerning the anxiety score, when comparing meditation intervention with control.

When performing a leave-one-out sensitivity analysis, the removal of any of the selected studies maintained the significance of the results and their direction favouring the meditation group (Figure 6).

**Figure 7:**
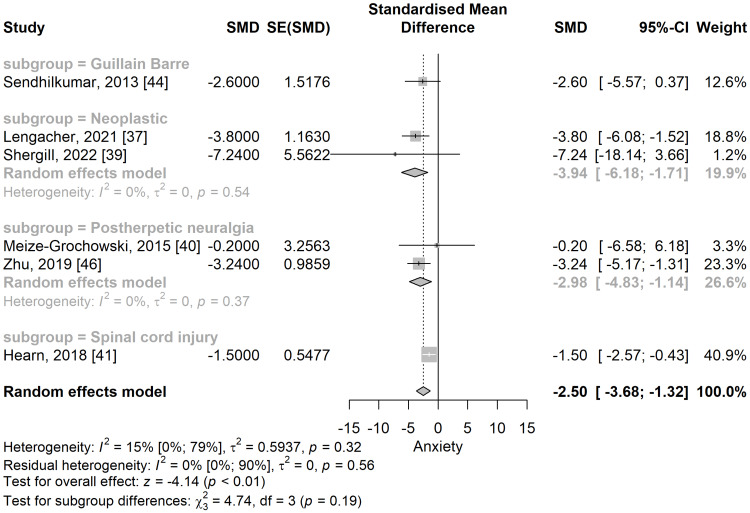
Standardized mean difference concerning the anxiety score, when comparing meditation intervention with control, and subgroup analyses in the function of the type of neuropathic pain. SMD: standardized mean difference; SE: standard error of the treatment effect; CI: confidence interval

In the subgroup analysis (Figure 7), post-herpetic neuralgia [40,46] (SMD: -2.98; 95% CI: -4.83 to -1.14), neoplastic [37,39] (SMD: -3.94; 95% CI: -6.18 to -1.71), and spinal cord injury [41] (SMD: -1.5; 95% CI: -2.57 to -0.43), anxiety scores were significantly lower in the meditation group compared to the control group.

**Figure 8:**
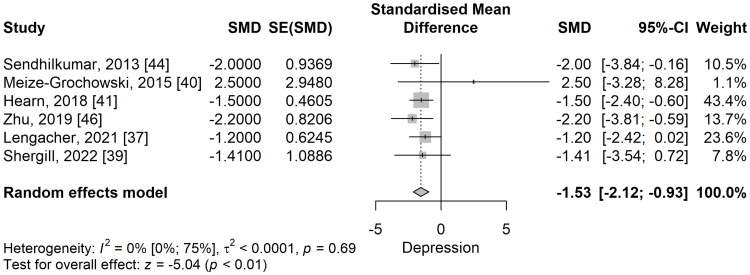
The standardized mean difference concerning the depression scores when comparing meditation intervention with control. SMD: standardized mean difference; SE: standard error of the treatment effect; CI: confidence interval

Depression: The depression scores were significantly lower in the meditation group compared to the control group in a meta-analysis of five studies (SMD: -1.53; 95% CI: -2.12 to -0.93; p=<0.001) (Figure 8). No significant heterogeneity was observed, I^2^ being 0% (95% CI: 0% to 74.6%), p=0.687.

**Figure 9:**
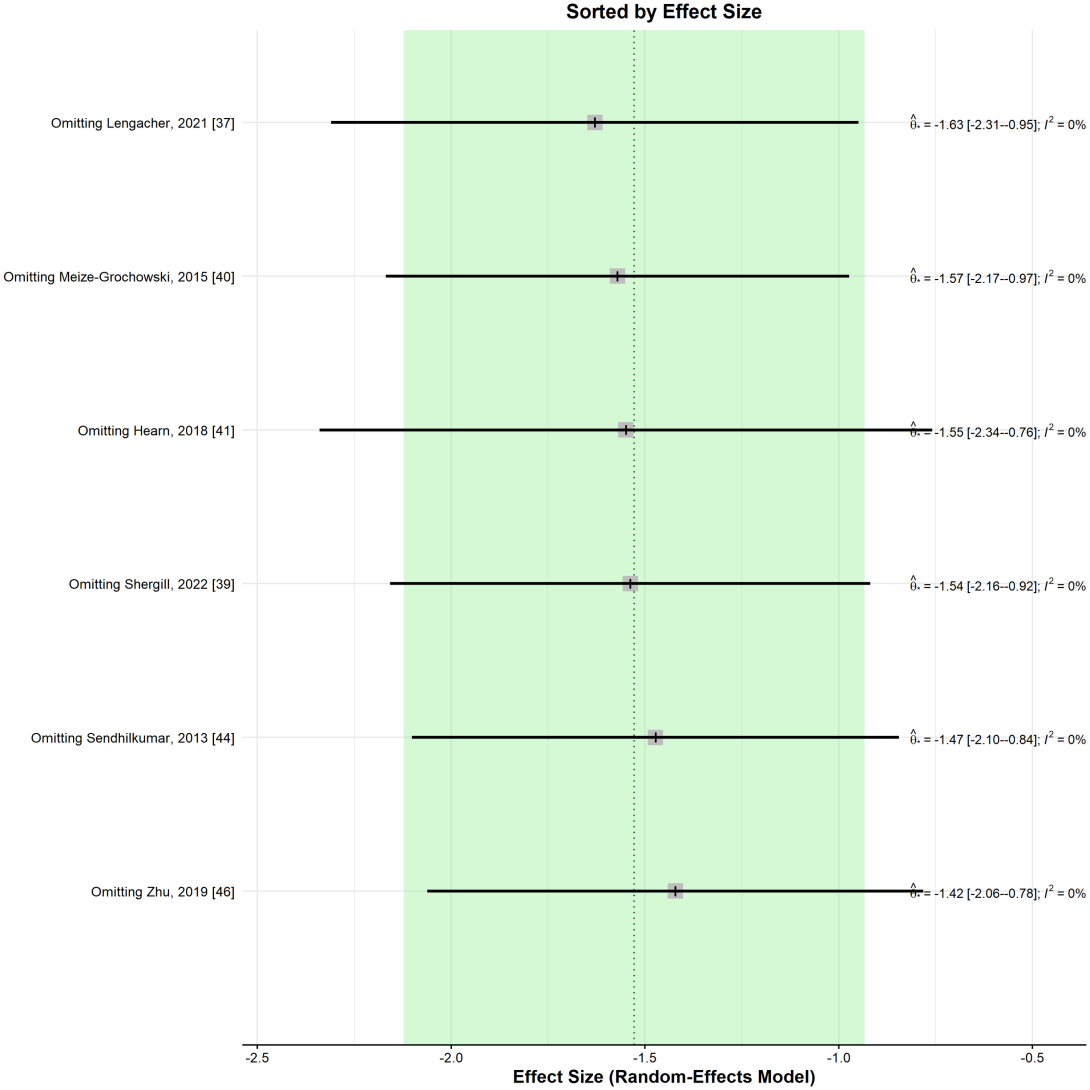
Leave-one-out sensitivity analysis plot for standardized mean difference (Θ ^[95% confidence interval]) concerning the depression score, when comparing meditation intervention with control.

When performing a leave-one-out sensitivity analysis, removing any of the selected studies maintained the significance of the results and their direction favouring the meditation group (Figure 9).

**Figure 10:**
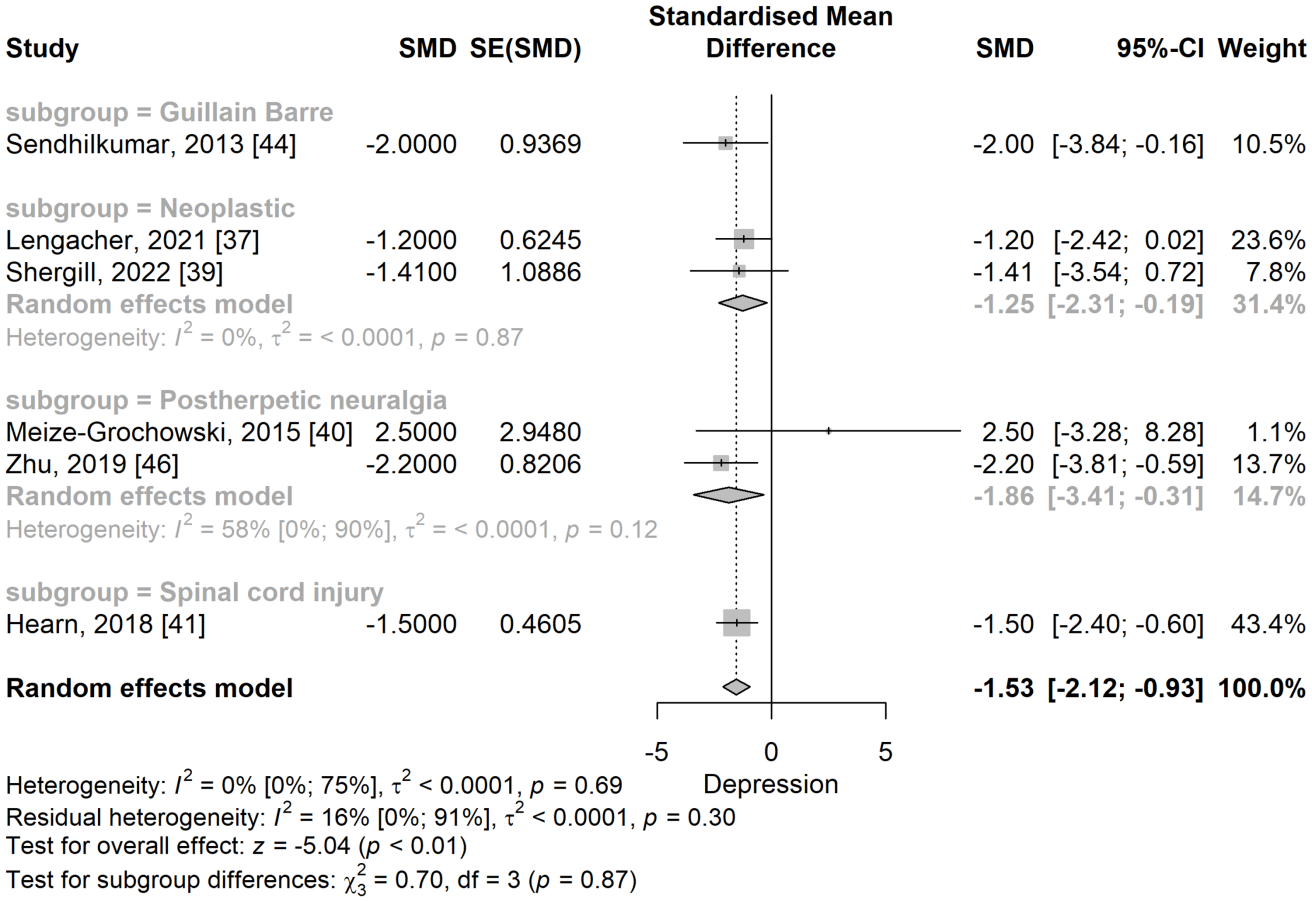
Standardized mean difference concerning the depression score, when comparing meditation intervention with control, and subgroup analyses in the function of the type of neuropathic pain. SMD: standardized mean difference; SE: standard error of the treatment effect; CI: confidence interval

In the subgroup analysis (Figure 10), post-herpetic neuralgia [40,46] (SMD: -1.86; 95% CI: -3.41 to -0.31), neoplastic [37,39] (SMD: -1.25; 95% CI: -2.31 to -0.19), spinal cord injury [41] (SMD: -1.5; 95% CI: -2.40 to -0.60), and Guillain-Barre syndrome (GBS) [44] (SMD: -2; 95% CI: -3.84 to -0.16), depression scores were significantly lower in the meditation group compared to the control group.

**Figure 11:**
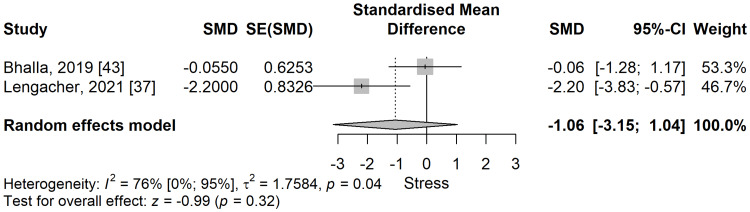
Standardized mean difference concerning the perceived stress (PS) scores when comparing meditation intervention with control. SMD: standardized mean difference; SE: standard error of the treatment effect; CI: confidence interval

Perceived stress: The perceived stress scores (PSS) were lower in the meditation group compared to the control group in a meta-analysis of two studies [37,43] but did not pass the threshold of significance (SMD: -1.06; 95% CI: -3.15 to 1.04; p=0.323) (Figure 11). The heterogeneity was substantial and statistically significant, with an I^2^ of 76% (95% CI: 0% to 95%), p=0.04.

**Figure 12:**
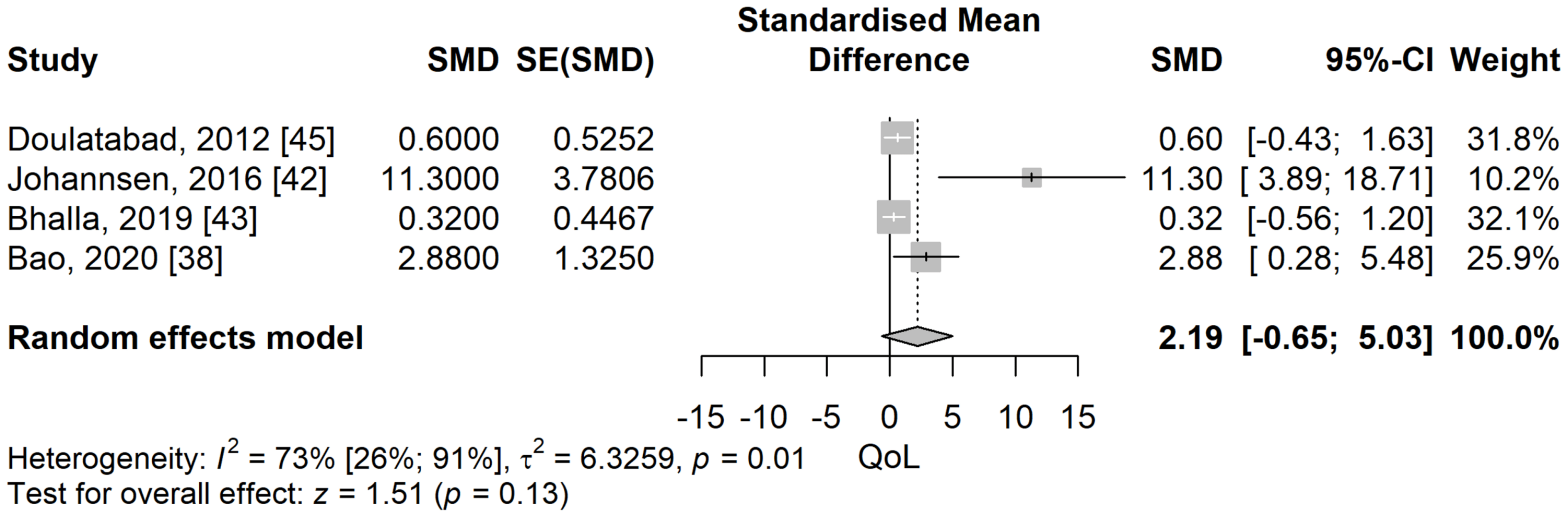
Standardized mean difference concerning the quality of life (QoL) scores when comparing meditation intervention with control. SMD: standardized mean difference; SE: standard error of the treatment effect; CI: confidence interval

Quality of life: The quality-of-life scores were higher in the meditation group compared to the control group in a meta-analysis of five studies but did not pass the threshold of significance (SMD: 2.19; 95% CI: -0.65 to 5.03; p=0.13) (Figure 12). The heterogeneity was substantial and statistically significant, with an I^2^ of 73.5% (95% CI: 25.6% to 90.6%), p=0.01.

**Figure 13:**
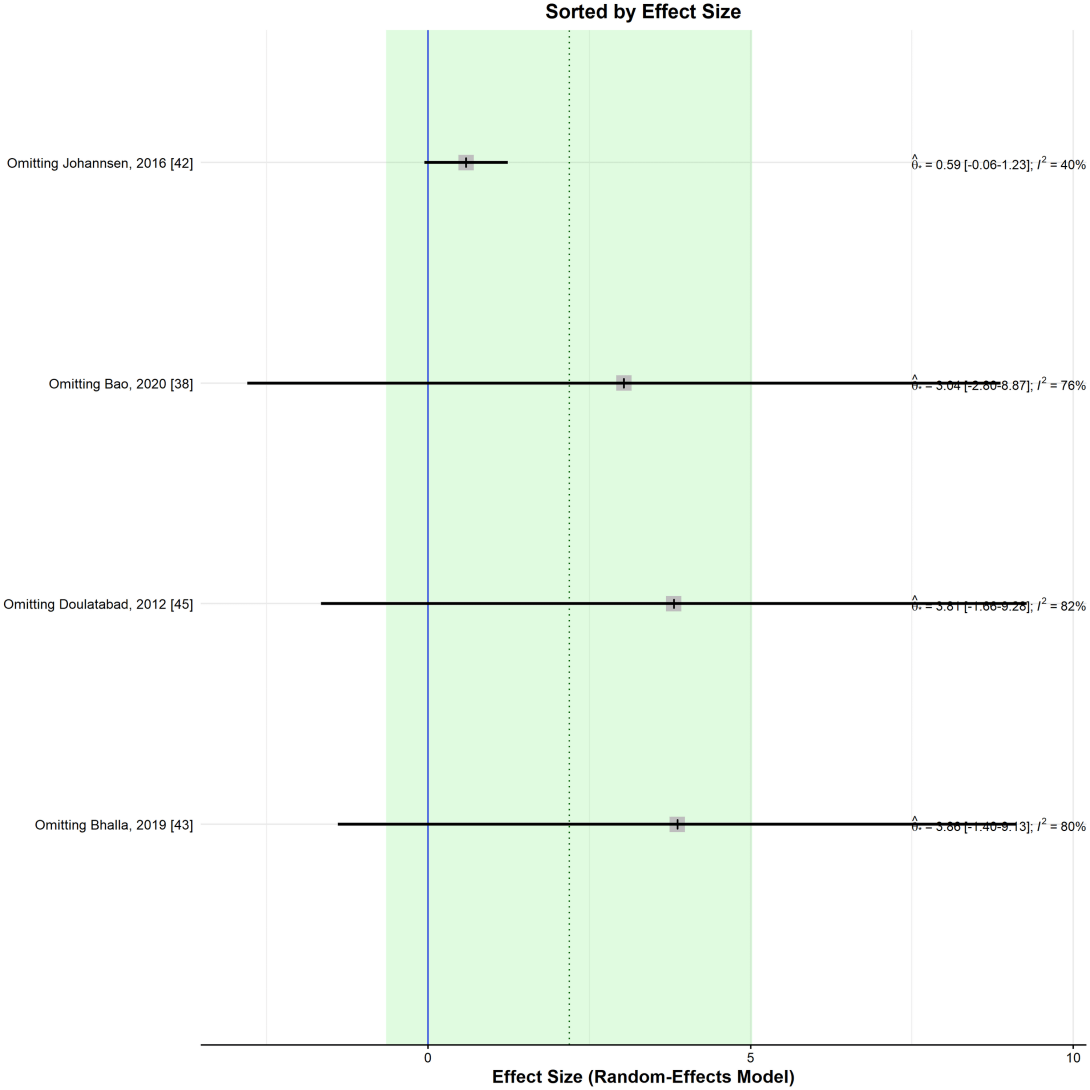
Leave-one-out sensitivity analysis plot for standardized mean difference (Θ ^[95% confidence interval]) concerning the quality-of-life score, when comparing meditation intervention with control.

Nevertheless, for all leave-one-out sensitivity analyses, the direction of the effect was in favour of the meditation group, albeit not statistically significant (Figure 13). For all possible subgroup analyses (MS [45], orofacial neuropathy [43], and neoplastic [38,42]), no significant differences were found between the two groups.

**Figure 14:**
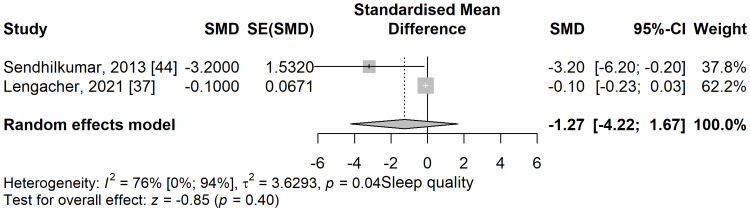
Standardized mean difference concerning the sleep quality scores, when comparing meditation intervention with control. SMD: standardized mean difference; SE: standard error of the treatment effect; CI: confidence interval

Sleep quality: The sleep quality scores were higher in the meditation group compared to the control group in a meta-analysis of two studies but did not reach the statistical significance level (SMD: -1.27; 95% CI: -4.22 to 1.67; p=0.397) (Figure 14). The heterogeneity was substantial and statistically significant, with an I^2^ of 76% (95% CI: 0% to 94%), p=0.04.

**Figure 15:**
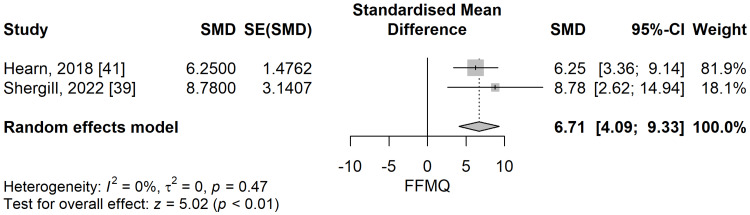
The standardized mean difference concerning the Five Facet Mindfulness Questionnaire (FFMQ) scores when comparing meditation intervention with control. SMD: standardized mean difference; SE: standard error of the treatment effect; CI: confidence interval

Mindfulness score: The mindfulness total scores, assessed by the Five Facet Mindfulness Questionnaire (FFMQ), were significantly higher in the meditation group compared to the control group in a meta-analysis of two studies (SMD: 6.71; 95% CI: 4.09 to 9.33; p=<0.001) (Figure 15). The heterogeneity, in turn, might not be important, considering I^2^ of 0%, p=0.47.

Follow-Up Measurements

**Figure 16:**
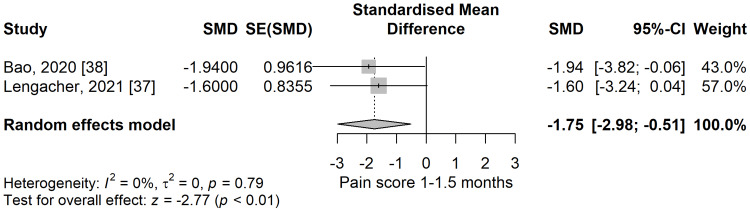
Standardized mean difference concerning the pain scores at 1-1.5 months follow-up when comparing meditation intervention with control. SMD: standardized mean difference; SE: standard error of the treatment effect; CI: confidence interval

Pain score 1-1.5 months: We found significantly lower pain scores in the meditation group compared to the control group (SMD = -1.75; 95% CI: -2.98 to -0.51; p=0.006) (Figure 16), at 1-1.5 months follow-up after the end of the study intervention. There was no heterogeneity, the values of I^2^ being 0%, p=0.79.

**Figure 17:**
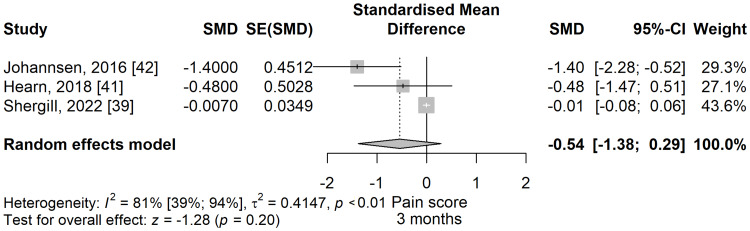
Standardized mean difference concerning the pain scores at three months follow-up, when comparing meditation intervention with control. SMD: standardized mean difference; SE: standard error of the treatment effect; CI: confidence interval

Pain score three months: The pain scores in the meditation group were lower compared to the control group (SMD = -0.54; 95% CI: -1.38 to 0.29; p=0.202) (Figure 17), at three months follow-up after the end of the study intervention, without reaching the level of significance. There was considerable heterogeneity, with I^2^ being 80.6% (95% CI: 39.2% to 93.8%), p=0.006.

**Figure 18:**
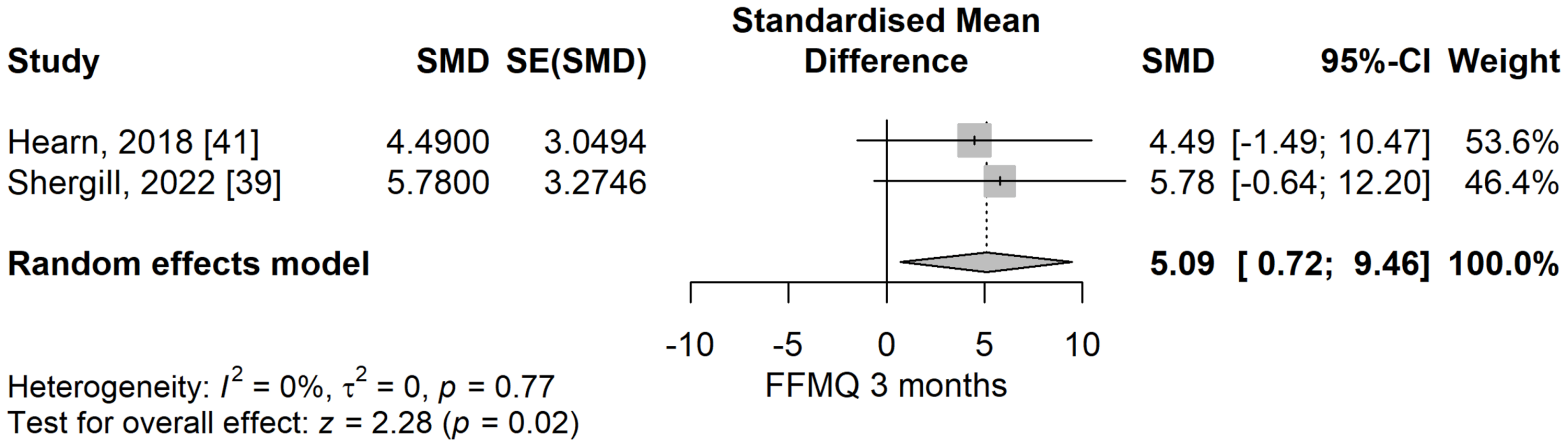
The standardized mean difference concerning the Five Facet Mindfulness Questionnaire (FFMQ) scores at three months follow-up when comparing meditation intervention with control. SMD: standardized mean difference; SE: standard error of the treatment effect; CI: confidence interval

Mindfulness score: The mindfulness total scores, assessed by the FFMQ scores, were significantly higher in the meditation group compared to the control group in a meta-analysis of two studies (SMD: 5.09; 95% CI: 0.72 to 9.46; p=0.023) (Figure 18). The heterogeneity, in turn, might not be important considering I^2^ of 0%, p=0.77.

Adverse Effects

Eight studies (80%) reported no information about side effects [37,40,41-46]. One study (Shergill et al., 2022 [39]) noted the absence of adverse effects, while another one (Bao et al., 2020 [38]) noted three out of 21 participants in a yoga group, with myalgia or pain.

Quality Assessment

**Figure 19:**
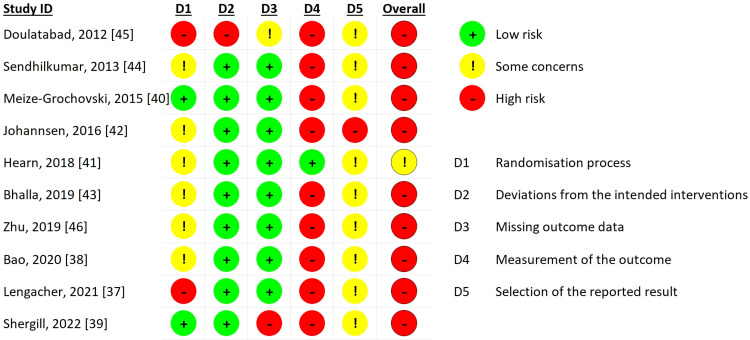
Risk of bias assessment of selected randomized controlled studies, using the Cochrane Risk of bias tool 2.

Using the Cochrane Risk of Bias tool 2, we assessed the methodological quality of the selected studies (Figure 19). Considering the randomization process domain, two studies (20%) were at high risk of bias, especially due to imbalances at baseline after randomization (Doulatabad et al., 2012 [45], Lengacher et al., 2021 [37]), while six studies (60%) (Bao et al. [38], Hearn et al. [41], Johanssen [42], Bhalla [43], Sendhikumar [44], Zhu [46]) had some concerns of bias, due to the absence of information on allocation concealment, and two studies (20%) were at low risk of bias (Meize-Grochowski et al., 2015 [40], Shergill et al., 2022 [39]). We found a high risk of deviation from the intended interventions domain only for one study (10%), since there was no transparency reporting intention to treat analysis (Doulatabad et al., 2012 [45]), all the other nine (90%) of the studies being at low risk of bias. There were missing outcome data, either not reported (Doulatabad et al., 2012 [45]), or important (Shergill et al., 2022 [39]) in two studies, thus indicating some concerns or high risk or bias, the other eight (80%) of the studies being at low risk of bias for this domain. In respect of the measurement of the outcome domain, nine (90%) studies [37-40,42-46] had a high risk of bias since, there are participant-reported outcomes, like pain, and they were not masked to the intervention, the outcome is subjective, and this is likely to influence the assessment of the outcome. Only one study [41] did not inform the participants that there was another possible intervention, thus this study was at low risk or bias for this domain. The measurements of the outcomes were unbiased, being either a numeric pain rating scale, or validated questionnaires for pain, depression and other endpoints. Six (60%) of the studies [37,40,41,43,44,46] did not mention a previous record of a research protocol. Although the rest of the four studies (40%) [38,39,42,44] had a research protocol, there were no clearly pre-specified analysis plans. Because of this, nine (90%) studies [37-41,44-46] were considered as having some concerns or bias regarding the selection of the reported result domain. For the same domain, one study (10%), Johannsen et al. [42], was considered at high risk or bias, since there were multiple eligible outcome measurements, the other studies having only one measurement per outcome. There were no issues about having multiple analyses for the same outcome. Overall, nine (90%) of the studies [37-40,42-46] were considered to be at high risk of bias, and one study [41] (10%) was considered to have some concerns of bias.

Questionnaires' Translation and Validity

Four studies used the questionnaires in the original language, American English [37-39,41], while the others, which were performed in Denmark, Iran, India, Mexico, and China used translations of the questionnaires, yet only one clearly stated the validation of the translated version of questionnaires [45]. For internal consistency, some of the studies presented Cronbach's alpha [40].

Publication Bias

**Figure 20:**
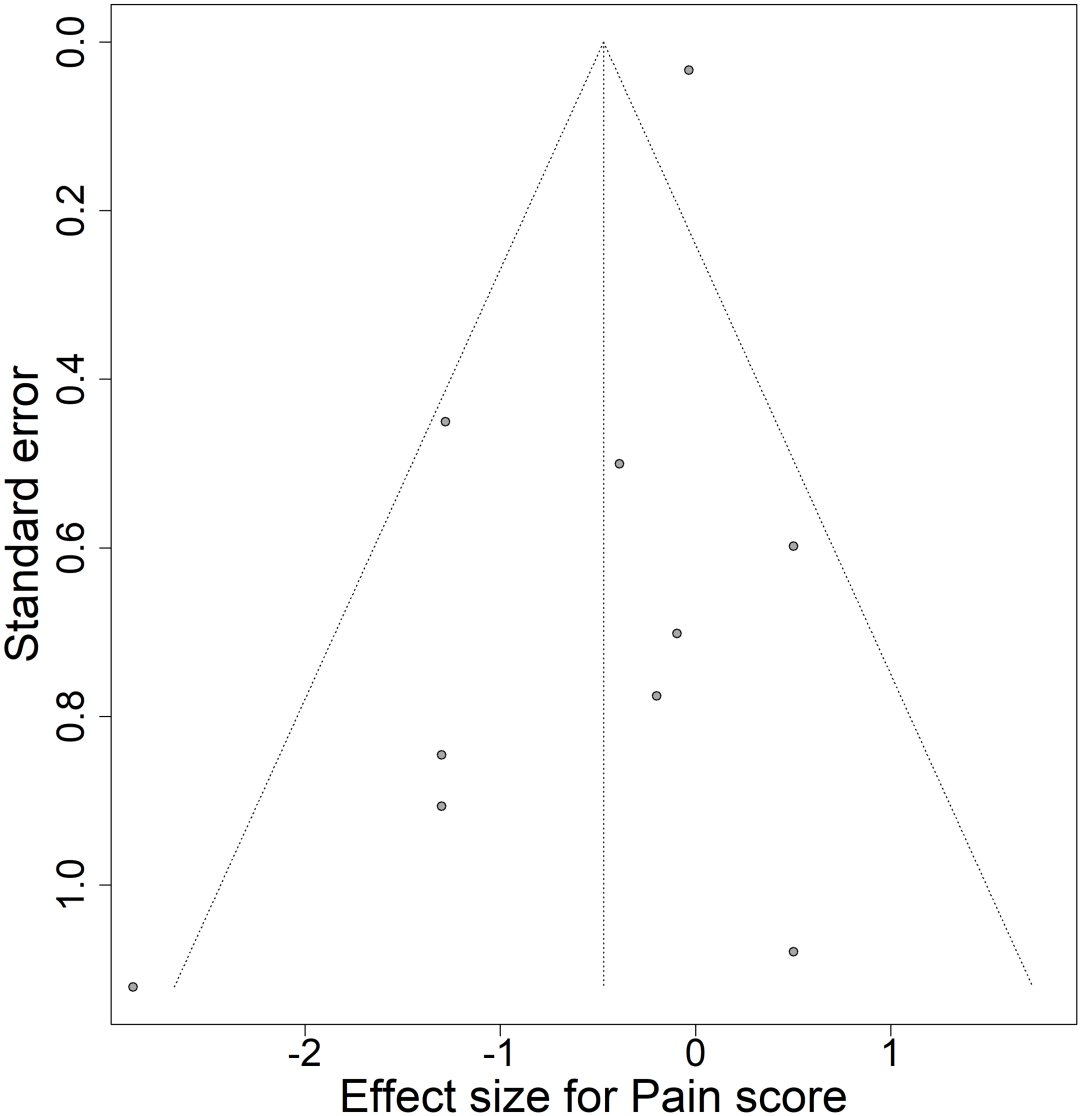
Funnel plot for Pain score, comparing Meditation with Control.

Publication bias was assessed for 10 studies concerning the pain scores at the end of the intervention period. No signs of asymmetry were observed in the funnel plot (Figure 20), and the p-value of the Egger test was 0.079. Thus no suggestion of publication bias. Similar assessments, yet for a smaller number of studies, were made, concerning anxiety, depression, quality of life, and sleep quality. For all these assessments, the p-value for the Egger test was above the threshold of significance.

Discussions

Main Findings

The systematic search conducted across seven databases identified 10 RCTs comparing mindfulness with control in subjects with chronic neuropathy, achieving its objectives. Concerning the main outcome, the pain severity score on all 10 studies meta-analyzed was lower in the mindfulness group, close to statistical significance; however, at 1-1.5 months follow up, on two studies [37,38] the difference was statistically significant; and then lost its significance at three months follow-up. The quality of life scores were higher in the meditation group compared to the control group, in the meta-analysis of four studies [38,42,43,45], but did not pass the threshold of significance. The anxiety, as well as the depression score, in the meta-analysis of six studies [37,39-41,44,46], was significantly lower in the meditation group compared to the control group. The PSS assessed in two studies [37,43] was lower in the meditation group compared to the control group without reaching a statistical significance level. The sleep quality score on a two study [37,44] meta-analysis achieved an improvement in the meditation group, yet did not reach the threshold of statistical significance. The mindfulness score in a two study [39,41] meta-analysis showed a significant increase in mindfulness score, in the intervention group compared to the control one, at the end of the intervention, and also at three months follow-up.

Subgroup Analyses

The subgroup analysis based on the type of neuropathic pain can provide valuable insights into the differential effects of mindfulness-based interventions across various neuropathic conditions.

Concerning the pain outcome, the majority of the studies showed lower pain scores in the meditation group compared to the control group. This is reflected in the same direction of result in the meta-analyses on almost all types of neuropathic pain: Guillain Barre, neoplastic, orofacial neuropathy, postherpetic neuralgia, and spinal cord injury. These results provide support to the idea that MBTs are capable of changing how people perceive pain in a variety of neuropathic diseases. This effect's consistency raises the possibility that mindfulness may be used broadly as a supplemental strategy to treat various neuropathic pain types. One exception was observed for MS where one study found higher pain scores in the meditation group compared to the control group. One explanation for this surprising finding could be the complex nature of MS-related pain, which frequently includes tiredness, stiffness, and cognitive problems that may interact differently with mindfulness techniques. Also, it's possible that increased awareness during mindfulness sessions heightened pain perception in these patients. Alternatively, the study sample may have had characteristics like different baseline pain, psychological distress, or medication use that altered the outcomes, or the mindfulness intervention may not have been well-suited to the unique needs of MS patients.

The anxiety subgroup analysis offers additional evidence of the potential advantages of mindfulness-based treatment methods for the psychological components of chronic neuropathic pain. The results show that patients in the meditation groups had significantly lower anxiety scores than those in the control groups for three specific neuropathic conditions: spinal cord injury, neoplastic neuropathy, and post-herpetic neuralgia. Persistent anxiety is frequently present in patients with chronic neuropathic pain, which may worsen pain perception and have a negative impact on quality of life. The capacity of mindfulness to improve emotional regulation, diminish rumination, and improve present-moment awareness - all essential elements in anxiety management - presumably accounts for its beneficial effects in these groups. Compared to other chronic neuropathic illnesses, for GBS, the result did not reach the significance level. This might be explained by the fact the disease has a relatively acute onset and a different disease trajectory, which may affect how well mindfulness therapies work. Furthermore, it's possible that the GBS sample size was smaller, which would have decreased the statistical power to identify significant differences.

Assessment of Heterogeneity

Some of the results were characterized by a substantial, statistically significant heterogeneity (the scores for pain and quality of life, sleep quality). Nevertheless, a major part of the results pointed in the same direction, the improving symptomatology of meditation-based therapy in the intervention arm.

Assessment of Measuring Instruments

The outcomes found in the evaluated studies were measured using certain validated scales. To assess the main outcome and intensity of pain, the following scales were used: Numerical Rating Scale (NRS) in four studies [38,41,44,46], brief pain inventory (BPI) in two studies [37,39], Short-Form McGill Pain Questionnaire (SF-MPQ-2) in two studies [40,42], and visual analogue scale (VAS) [43], Multiple Sclerosis Quality of Life-54 (MSQoL-54) [45], each in one study. NRS is an 11-point numerical scale, which has been shown to be a sensitive and reliable pain measure [53]. The BPI scores are presented as providing broader information about the patient outcome and pain-related disability compared with pain severity scores alone [54] and constitute a main outcome recommended by the Initiative of Methods, Measurement, and Pain Assessment in Clinical Trials (IMMPACT) group [55]. VAS is a standardized visual analogue scale. The SF-MFQ-2 offers composite and subscale measures of the major sensory and affective items (e.g., fearful, tiring-exhausting, sickening, etc.) for both neuropathic and non-neuropathic pain. The total and subscale scores of the SF-MPQ-2 have shown change-responsiveness and were found to be meaningful to patients [56]. The MSQOL-54 is a multidimensional health-related quality-of-life measure that combines both generic and MS-specific items into a single instrument. This 54-item instrument generates 12 subscales (among them pain, quality of life, emotional well-being, etc.) along with two summary scores, and two additional single-item measures [57].

The quality of life was assessed using different validated instruments which are presented briefly below. MSQoL, used in one study [45], measures as mentioned above, multiple items through certain subscales. Among them, there are pain and quality of life, the ones of interest for the present review. World Health Organization-5 Wellbeing Index (WHO-5) was used in one study [42]. It measures psychological well-being and studies have found it expresses aspects other than just the absence of depressive symptoms [58]. Short-Form-12 Health Survey (SF-12v.2) was used in one study [39]. It is a brief, 12-item self-report measure of health-related quality of life and it takes a few minutes to complete it. It is based on the well-known and empirically validated Short-Form-36 [59]. The functional assessment of cancer therapy/gynecologic oncology group neurotoxicity (FACT/GOG-Ntx) questionnaire as an 11-item Neurotoxicity subscale was used in one study [38]. It assesses neuropathy-related quality of life. It demonstrated clinical validity and sensitivity to longitudinal symptom changes and addresses sensory, motor, and auditory neuropathy, and dysfunction associated with neuropathy [60]. The Medical Outcomes Study Short Form (MOS SF-36), also named the RAND 36-Item Health Survey 1.0, was used in two of the studies [37,40]. It assesses mental and physical health as related to QoL; is a broad health questionnaire that produces an eight-scale profile of physical functioning, bodily pain, role limitations due to physical health problems, role limitations due to personal or emotional problems, emotional well-being, social functioning, energy/fatigue, and general health perceptions; higher scores have shown better mental and physical health [61]. World Health Organization Quality of Life (WHOQoL- BREF) was used in one of the studies [41]. It is a 26-item questionnaire and measures QoL in four domains, graded on a five-point Likert scale: physical health, psychological health, social relationships and environment [62]. The American Chronic Pain Association's quality of life scale (ACPA QoL) is a one-item assessment of function for people with chronic pain. Quality of life is assessed on an 11-point scale ranging from "Non-Functioning" to "Normal Quality of Life". The ACPA Quality of Life Scale was created primarily to assess functioning in people with chronic pain [63].

To assess anxiety four different instruments were used: the Hamilton Anxiety Rating Scale (HAMA), the Hospital Anxiety and Depression Scale (HADS), the Profile of mood states (POMS) and the State-Trait Anxiety Inventory. The HAMA was designed by Hamilton in 1959 and used to measure the anxiety level in neurosis and other patients, comprising 14 items. In HAMA, all items use a five-point scale [64]. The HADS is a 14-item Likert scale measure; seven items assess the severity of depression and seven items assess the severity of anxiety, and responses range from 0 to 3 [65]. Higher scores (range 0 to 21 on each outcome) indicate greater symptom severity. The HADS total score has shown good psychometric qualities as an overall measure of distress [66]. The POMS assesses six aspects of mood. The scores are related to tension-anxiety, depression-dejection, anger-hostility, vigour, exhaustion and perplexity. It has been applied frequently to medical and psychiatric populations. The POMS evaluates state (as opposed to trait) characteristics, making it suitable for repeated measurements [67]. The State-Trait Anxiety Inventory-State, a subscale of the State-Trait Anxiety Inventory, was used to measure situational anxiety, in one of the studies; higher scores are indicative of greater anxiety [68].

In order to assess depression, four instruments were used: the Hamilton Depression Rating Scale (HAMD), HADS, Patient Health Questionnaire-9 (PHQ-9), and the Center for Epidemiologic Studies Depression Scale. The HAMA was created by Hamilton in 1960 (Hamilton, 1960; Hamilton, 1967). It uses 24 items to evaluate the level of depression. Among them, 14 items use a five-point scale, and another 10 items use a three-point scale; if the total score of the HAMD is higher than 18, the patient will be considered carrying depression [69]. The PHQ-9 is a nine-item scale which assesses the severity of depressive symptoms over the past two weeks. It is based on the Diagnostic and Statistical Manual of Mental Disorders, fourth edition (DSM-IV) diagnostic criteria for major depression [70]. This questionnaire is ranked from 0 to 27, with clinical cut-points indicative of mild, moderate, moderately severe, and severe depression. A five-point decrease in the PHQ-9 is considered to be the minimum clinically significant progress [71].

For psychological distress assessment, two scales were used: PSS and the American Heart Association Stress Scale. The PSS was created to quantify how stressful people perceive their daily circumstances to be. The PSS is recommended as an outcome measure of experienced levels of stress [72]. The PSS has been used among individuals with pain and the psychometric properties of the instrument are well-documented and demonstrate good reliability, internal consistency, and discriminant validity [72].

The sleep quality assessment was effected using the Pittsburg Sleep Quality Index (PSQI). It evaluates the quality of sleep in the previous two weeks through seven items: subjective sleep quality, sleep latency, sleep duration, habitual sleep efficiency, sleep disturbances, use of sleeping medication, and daytime dysfunction [73]. It is a self-rated scale. The scoring of answers is based on a 0-3 scale, whereby 3 reflects the negative extreme on the Likert Scale. The responses are added to give a composite global PSQI [73], score. A poor quality of sleep means a global sum of "5" or greater.

For the mindfulness assessment, the FFMQ was used. FFMQ is a 39-question instrument referring to five aspects of mindfulness: "non-reactivity to inner experience, observing, describing, acting with awareness, and non-judging of experience" [74]. Participants are rating statements, using a five-point Likert-type scale, regarding their truthfulness, as they perceive it. The FFMQ has been shown adequate to good reliability, with alpha coefficients ranging from 0.75 to 0.91 for all subscales [29]. Mindfulness total score may suggest a significant proportion of the variance in pain catastrophizing, pain-related fear, pain hypervigilance, and disability [29].

Comparison With Other Systematic Reviews

Referring to the results on the same topic as the one of the present systematic review and meta-analysis, the current literature presents shared conclusions. In a systematic review by Ronconi et al. (2024) [75], on the effect of non-pharmacological treatments for chemotherapy-induced peripheral neuropathies (CIPN), it is shown yoga appears as a safe and good remedy for depression, fatigue, and sleep impairment in patients with breast cancer, however, there is no direct evidence of efficacy for CIPN symptoms. Papadopoulou et al. (2023) revealed in a meta-analysis on non-pharmacological interventions and CIPN, that yoga improved the pain severity score in a statistically significant manner, yet not the quality of life [76]. In a meta-analysis of Bhardwaj et al. (2023) [77] on yoga and neuropathic pain, it was revealed that yoga had an overall positive effect on NP, albeit not statistically significant. It emphasizes yoga's promising role in reducing pain intensity and improving quality of life in NP disorders, while also being a low-cost and easily accessible modality of therapy. Moisset et al. (2020) emphasized in a systematic review of neuropathic pain and pharmacological and non-pharmacological therapies, constituting in French guidelines recommendations, that cognitive behavioural therapy (CBT) and mindfulness could be used as second-line treatments in addition to other therapies, due to their safeness, and high levels of acceptability and feasibility, even in children or elderly patients [78].

Cultural Differences

It was noticed none of the included studies addressed the subject of the cultural differences in mental-based therapies, as a distinctiveness element. The influence of cultural differences on the outcomes of meditation-based therapies for chronic neuropathic pain is a specific factor that should be considered. Cultural norms and beliefs about pain, as well as attitudes towards meditation, can vary significantly across different populations, potentially impacting both the perception of pain and the effectiveness of meditation-based interventions. Cultural differences in pain expression and tolerance are well-documented, with some cultures emphasizing stoicism while others encourage more expressive reactions to pain [79,80].

Cultural attitudes toward meditation itself can also vary. In an article from Jon Kabat-Zinn (2003) [81], on mindfulness in context, it is shown cross-cultural and paradigm issues need to be addressed, with intimate sensitivity. In a review published by Castellanos et al. (2019) [82] it is revealed there is clear evidence that cultural adaptations can improve evidence-based treatment implementation among Hispanics (the fastest-growing cultural minority from the USA). A recent study, by Listyandini et al. (2023) [83], was conducted on culturally adapting an internet-delivered mindfulness program to Indonesian college students with psychological distress. It highlighted the importance of the cultural adaptation of an evidence-based mindfulness intervention and the study found this culturally adapted program was relevant for Indonesian students.

These factors underscore the need for culturally sensitive approaches when implementing meditation-based therapies across diverse populations. This is to ensure, from this point of view, too, the right conditions in order to achieve the effectiveness of such interventions.

Study Limitations

There are several limitations to be acknowledged. The risk of bias in the selected studies represents one of them. Since in most of the studies the participants were not masked to the intervention, and they reported the outcomes which were subjective, even if the assessors were blind to the intervention, the measurement of the outcome domain was at high risk of bias. Knowing their intervention, they might report the intervention to be either better or worse than it was, in the function of their trust or thoughts about the method, thus inducing a bias with unpredictable direction. One study prevented this by hiding the information about the other method [41]. This approach minimizes the bias but is questionable in the ethics realm. The next problematic domain was the randomization process where most of the studies were at least at high risk of bias, due to the unknown status of allocation concealment, or to imbalances in baseline characteristics. Here, it is possible that some studies did use allocation concealment, but the absence of reporting prevents the possibility of diminishing the risk or bias level. The third problematic domain was concerning the selection of the reported result, due to the absence of a clearly pre-specified analysis plan, in all the studies, thus indicating some concerns of bias. In reality, if the authors did think only about one variant of analysis, and used it, then the risk of bias would be low. It can be possible that most of the articles are in this scenario, and in a very likely way, but there was no transparency to ascertain this. Another shortcoming was the small number of subjects (20-40 patients) involved in some studies [27,29,32,33]; nevertheless, this kind of review helps to limit this by assessing more studies at once. Even so, some of the results were statistically significant and were robust to sensitivity leave-one-out analyses. The questionnaires used in this review as outcomes were originally developed in American English. Four studies were carried out in English-speaking countries - USA [37,38], Canada [39], UK [41] and the rest in other five countries - Mexico [40], Denmark [42], India [43,44], Iran [45] and China [46]. These latter studies used translations of the questionnaires and only one clearly stated the validation of the translated version. For internal consistency, some of the studies presented good Cronbach's alpha for the validated questionnaires. Using questionnaires in different languages can introduce a measurement bias and implicit comparability issues between studies, even for validated instruments. There are some sources of clinical heterogeneity between the studies: with respect to the diseases - yet all involve pain and mood and emotional involvement; the variants of mediation interventions - nevertheless all have at their core the same principles; the length of the intervention - yet the majority were of at least six weeks, usually eight weeks; the measurement instruments - but usually most used the same type of instrument, or equivalent ones, or very similar ones, and furthermore, the SMD allows to pool the results together in these situations. Moreover, the observations were performed in different countries and cultures, that might respond differently to the interventions and induce heterogeneity in the results. Still, regarding this clinical heterogeneity of neuropathic diseases, pain in neuropathies is now widely accepted as having multiple etiologies across similar neuropathic syndromes [84]. The method of researching numerous neuropathic pain etiologies appears useful, as the therapy of chronic neuropathy focuses on symptomatology rather than aetiology [85].

Future Directions

The limitations that were found may constitute the ground for future improvement in meditation studies. The simplest measures to be implemented would be the methodological rigour to minimize the risk of bias, by the use of allocation concealment (that can be always used in trials), by clear use and reporting of the intention-to-treat analysis, by a transparent pre-specified analysis plan, by a clear reporting of patients lost to follow-up, with their motives. Improvements should be made to make extensive use of stratified randomization, or minimization randomization methods, for known important confounders (like the disease stage, or subtypes, pain levels, depression levels, anxiety levels) to improve the similitude of baseline characteristics. An important issue that was rarely addressed was the quantification of the use of pain medication (or medication for other outcomes) in both groups and moreover an accounting of it with statistical analyses. Due to the fact meditation is not a mainstream intervention, it can be approached with mistrust, and then the treatment adherence can be low, and influence the results. Thus, the belief in this meditation intervention and treatment adherence should be measured strictly in future studies. Most of the studies did not mention the presence of side effects during the intervention [37,40,41-46]. There are studies showing some adverse effects during meditation practice, like anxiety, depression, or other negative impact on daily life [86,87]. Therefore, it is recommended that future studies on meditation systematically document and report adverse events, and whether these occur.

Study Strengths

In addition, the present review has the following strengths: (1) The Cochrane Collaboration's Risk of Bias Tool, version 2, from one of the most prestigious organizations that conducts systematic reviews and develops high-quality instruments for study validity evaluation, was used to evaluate the publications' methodological flaws. (2) A comprehensive search strategy was used. (3) Many databases, more exactly seven (PubMed, EMBASE, Cochrane Database, Scopus, Web of Science, PsycheNet, Lilacs), were searched. (4) Only RCTs were included. (5) Sensitivity analyses and subgroup analyses were performed. (6) This review assesses not one but several different types of meditation-based interventions, giving a broader perspective on such therapies in chronic neuropathy. (7) The approach of studying multiple neuropathic pain etiologies seems beneficial as the treatment of chronic neuropathy addresses rather its symptomatology than aetiology [85]. (8) The review presents a discussion on the potential impact of cultural differences on the study outcomes. (9) The article explores the specific mechanisms by which these therapies might achieve these effects. In the context of chronic neuropathic pain, it presents the impact on specific brain regions, neurotransmitter modulation, potential for neuroplasticity and also, the psychological mechanisms.

Clinical Implications

MBTs could be integrated into existing treatment protocols as a complementary approach, particularly for addressing pain, anxiety, and depression in chronic neuropathy patients. From a clinical perspective, the integration of therapies based on meditation could start with organized programs like MBSR, which are taught by qualified instructors in both group and individual settings. These programs can be incorporated into multidisciplinary care regimens, giving patients useful tools to control their symptoms in addition to conventional treatments. Additionally, in order to enable patients to take an active role in their pain management, healthcare professionals might suggest shorter, daily meditation practices. Adoption of MBTs may also result in less dependence on medications, especially opioids and other analgesics, which lowers the possibility of side effects and enhances patient outcomes overall. As these therapies gain traction, they could be incorporated into clinical guidelines for chronic neuropathy. This will require additional research to discover the best ways to administer the therapies and what combinations of other treatments work best.

## Conclusions

The present systematic review with meta-analysis of RCTs found the pain score was significantly lower in the meditation group at 1-1.5 months follow up; the anxiety levels and depression scores were revealed to be significantly lower in the meditation group compared to the control one, and the mindfulness scores were significantly higher in the meditation group compared to the control one, at the end of the intervention, as well as at three months follow-up. The quality-of-life scores and the sleep quality scores were higher, while perceived stress was lower in the meditation group without reaching statistical significance. The analyzed trials, however, carry some methodological shortcomings, which affect the quality of the observed evidence. Substantial improvements in methodological quality will bring about more rigorously derived results in future studies.
